# Detecting destabilizing species in the phylogenetic backbone of *Potentilla* (Rosaceae) using low-copy nuclear markers

**DOI:** 10.1093/aobpla/plaa017

**Published:** 2020-05-09

**Authors:** Nannie L Persson, Ingrid Toresen, Heidi Lie Andersen, Jenny E E Smedmark, Torsten Eriksson

**Affiliations:** Department of Natural History, University Museum, University of Bergen, Postboks, Bergen, Norway

**Keywords:** Autopolyploidy, Fragarioides, incomplete lineage sorting, Multispecies Coalescent, Potentilleae

## Abstract

The genus *Potentilla* (Rosaceae) has been subjected to several phylogenetic studies, but resolving its evolutionary history has proven challenging. Previous analyses recovered six, informally named, groups: the Argentea, Ivesioid, Fragarioides, Reptans, Alba and Anserina clades, but the relationships among some of these clades differ between data sets. The Reptans clade, which includes the type species of *Potentilla*, has been noticed to shift position between plastid and nuclear ribosomal data sets. We studied this incongruence by analysing four low-copy nuclear markers, in addition to chloroplast and nuclear ribosomal data, with a set of Bayesian phylogenetic and Multispecies Coalescent (MSC) analyses. A selective taxon removal strategy demonstrated that the included representatives from the Fragarioides clade, *P. dickinsii* and *P. fragarioides*, were the main sources of the instability seen in the trees. The Fragarioides species showed different relationships in each gene tree, and were only supported as a monophyletic group in a single marker when the Reptans clade was excluded from the analysis. The incongruences could not be explained by allopolyploidy, but rather by homoploid hybridization, incomplete lineage sorting or taxon sampling effects. When *P. dickinsii* and *P. fragarioides* were removed from the data set, a fully resolved, supported backbone phylogeny of *Potentilla* was obtained in the MSC analysis. Additionally, indications of autopolyploid origins of the Reptans and Ivesioid clades were discovered in the low-copy gene trees.

## Teaser

It can be challenging to reconstruct more ancient branches in the tree of life, given complicating factors such as rapid speciation and hybridization between species. We addressed known challenges about relationships in the cinquefoils (genus *Potentilla*, strawberry-like herbs in the flowering plant family Rosaceae) by analysing DNA sequences from different regions of the genomes of representative species. We present a new well-supported phylogenetic tree that provides a fundamental tool for understanding the evolution of species and their traits in this plant group.

## Introduction

Polyploidy is a well-known and common phenomenon in plants, defined as having three or more complete sets of chromosomes. All extant species of flowering plants may in fact be paleopolyploids, as a result of whole-genome duplications early in the history of the angiosperms (Cui *et al.* 2006). However, through a number of different processes resulting in genomic reorganizations, many species with polyploidy in their ancestry now function as diploids (Leitch and Bennett 2004; Bento *et al.* 2011; Mandáková *et al.* 2017). The genus *Potentilla* (Rosaceae) consists of ~400 species which are mainly yellow-flowered, herbaceous perennials from the Northern Hemisphere. There are diploid as well as polyploid species (Index to Plant Chromosome Numbers, IPCN 1979; Kurtto *et al.* 2004), with ploidy levels of up to hexadecaploid (16*x*) (Kalkman 2004), and a base chromosome number of 7. Polyploidization as well as hybridization are considered important processes in the evolution of *Potentilla* (Potter *et al.* 2007; Dobeš and Paule 2010; Paule *et al.* 2011, 2012).

In the latest monograph of *Potentilla*, Wolf (1908) identified just over 300 species and divided them into six subsections based on style shape and its position on the ovary. Even though the first molecular studies of *Potentilla* showed that the genus was not monophyletic as circumscribed by Wolf (Eriksson *et al.* 1998, 2003), recent classifications maintain a non-monophyletic *Potentilla* by recognizing the genera *Horkelia*, *Horkeliella*, *Ivesia* and *Duchesnea* (Chaoluan *et al.* 2003; Ertter and Reveal 2014a, b; Kechaykin and Shmakov 2016). Although certain aspects of their morphology differ from most other *Potentilla* species, molecular studies have consistently shown that these genera are nested within the *Potentilla* clade (Eriksson *et al.* 1998, 2003; Dobeš and Paule 2010; Töpel *et al.* 2011; Feng *et al.* 2017; Xiang *et al.* 2017; Persson *et al.* 2020).

The phylogenetic study of *Potentilla* by Töpel *et al.* (2011), based on chloroplast and nuclear ribosomal data, identified six major clades that were informally named the Argentea, Ivesioid, Fragarioides, Reptans, Alba and Anserina clades. They found the style type character used by Wolf (1908) to be informative, largely corresponding to the different clades. Using the same type of molecular data, Dobeš and Paule (2010) and Feng *et al.* (2017) also recovered these clades. However, not all of the clades are well-supported, nor are the relationships between them certain. One of the most prominent incongruences concerns the Reptans clade and its position in relation to the Fragarioides clade. The Reptans clade includes the type species of *Potentilla*, *P. reptans*, and corresponds to ‘Grex’ Tormentillae in the monograph by Wolf (1908). It comprises eight species that are found in Europe, Asia and North America (Global Biodiversity Information Facility, GBIF Secretariat 2019), characterized by having long pedicels (Wolf 1908). All species but one are polyploid (IPCN) and they form a clade in previous phylogenetic analyses (Eriksson *et al.* 1998, 2003; Dobeš and Paule 2010; Töpel *et al.* 2011; Feng *et al.* 2017). Grex Fragarioides comprises, according to Wolf (1908), two species; *P. fragarioides* and *P. freyniana*, characterized by pinnate leaves where the three terminal leaflets are much larger than the proximal leaflets. Töpel *et al.* (2011) associated two additional species with this clade; *P. dickinsii* in Grex Eriocarpae, characterized by the indumentum of the fruits (Wolf 1908) and *P. stolonifera* (Grex Fragarioides, as *P. fragarioides* var. *stolonifera*). These four species are found in East Asia (GBIF) and are diploid according to published chromosome counts (IPCN).

Reconstructing species phylogenies with chloroplast DNA can be problematic with polyploids (and allopolyploids in particular), since chloroplast DNA is uniparentally inherited and therefore not able to recover polyploid signals. Similarly, nuclear ribosomal DNA is typically subject to concerted evolution with homogenization towards either the maternal or the paternal lineage (Wendel 2000). In certain cases, discrepancies seen between chloroplast and nuclear ribosomal phylogenies may be explained by hybridization and diversification of fertile hybrids or by allopolyploidization (Lundberg *et al.* 2009; Töpel *et al.* 2011). Low-copy nuclear (LCN) markers are better candidates for resolving relationships where the species are known to be polyploid. This is because subgenome-specific copies are, at least initially after a polyploidization event, present in each subgenome, inherited biparentally and less influenced by concerted evolution (Small *et al.* 2004). Several studies have used LCN markers to resolve phylogenetic relationships, and to trace polyploidization and hybridization events, at different taxonomic levels within Rosaceae, such as the Maloideae subfamily (Evans and Campbell 2002), subtribe Geinae (Smedmark *et al.* 2005), *Prunus* (Shi *et al.* 2013) and *Potentilla* (Persson *et al.* 2020). However, LCN markers have so far not been used to resolve the phylogenetic backbone structure of *Potentilla*. A robust backbone is of great benefit to future studies within *Potentilla*, as a basis for studies of historical biogeography or for classification. It can also be used to select proper outgroups when investigating internal relationships of the subclades. Lastly, certain flower and leaf characteristics have been used in classifications of tribe Potentilleae, and we need this backbone in order to more securely trace the evolution of such characteristics on the branches of the phylogeny.

The aim of this study is to (i) infer the backbone phylogeny of *Potentilla* and (ii) to identify underlying sources of incongruence between conflicting topologies. We present four gene trees based on LCN markers and compare our results with chloroplast and nuclear ribosomal phylogenies. In addition, two species trees are presented, showing a supported backbone after the sources of incongruence are removed.

## Materials and Methods

### Plant material

Twenty-four specimens from 19 species (including subspecies) were selected to represent the six major clades identified in recent studies of *Potentilla* (Dobeš and Paule 2010; Töpel *et al.* 2011; Feng *et al.* 2017), including species that have been classified in the genera *Horkelia*, *Horkeliella* and *Ivesia* of the Ivesioid clade (Ertter and Reveal 2014a), *Duchesnea* of the Reptans clade (Chaoluan *et al.* 2003; Ertter and Reveal 2014b) as *P. indica* in this study and *Argentina* and *Tylosperma* of the Anserina clade (Table 1). Plant material for DNA extraction was obtained from botanical gardens (Bergius Botanic Garden Stockholm, Bonn University Botanic Gardens, The Linnéan Gardens of Uppsala and Royal Botanic Garden Edinburgh) and herbaria (BG, E, GB, MARY, O, S and UPS).

**Table 1.** Taxa included in this study with clade affiliation, ploidy level, voucher and GenBank accession numbers. ^a^IPCN (and references within); ^b^Kurtto *et al.* (2004) (and references within); ^c^Baldwin *et al.* (2012).

				Accession number					
Taxon	Clade	Ploidy level	Voucher	matK	ITS	DHAR2	GAPCP1	GBSSI-2	SbeI
*Potentilla heptaphylla*	Argentea	2*x*, 4*x*^a,b^	Persson & Eriksson 28 (BG)	MT134122	MT112958	MT134200(C) MT134201(D) MT134202(E) MT134203(G)	MN346831(C) MN346832(E) MN346833(F)	MT134234(9) MT134235(14) MT134236(15)	MT134138
*Potentilla hirta*	Argentea	2*x*^a,b^	Cult. Royal Botanic Garden Edinburgh 1962-1846; BROWP 1238 (E)	MT134123	MT112959	MT134204	MN346834	MT134237	MT134139
*Horkelia bolanderi*	Ivesioid	–	Cult. Bergius Botanic Garden, Stockholm 5353 (no voucher)	–	MT112952	MT134186(A) MT134187(B) MT134188(E) MT134189(F)	MN346800(A) MT134151(7b) MT134152(15) MT134153(16)	MT134225(1) MT134226(10)	MT134133
*Horkelia californica*	Ivesioid	4*x*^c^	Balls 9326 (S)	MT134117	–	MT134190(B) MT134191(E) MT134192(H)	MN346803(H) MN346804(L)	–	–
*Horkeliella purpurascens*	Ivesioid	–	Eriksson 818 (S)	–	MT112966	–	MT134171(2) MT134172(2b) MT134173(3) MT134174(5b)	MT134252(1) MT134253(4)	MT134145
*Ivesia gordonii*	Ivesioid	–	Porter 6666 (UPS)	MT134121	–	MT134198(D) MT134199(E)	MN346808(C) MN346809(E) MN346810(H)	–	–
*Ivesia kingii* var. *eremica*	Ivesioid	–	Reveal *et al.* 4782 (MARY)	–	MT112962	MT134208(B) MT134209(E)	MN346812(B) MT134163(2) MT134164(7) MT134165(10)	MT134244(4) MT134245(7) MT134246(11)	MT134142
*Ivesia kingii*	Ivesioid	–	Mats Töpel 355 (GB)	FR851328	–	–	–	–	–
*Ivesia multifoliolata*	Ivesioid	–	Eriksson 820 (S)	MT134127	MT112965	MT134213(D) MT134214(G)	MN346813(A) MN346814(B) MT134169(1) MT134170(6)	MT134251(1)	MT134144
*Potentilla ancistrifolia* var. *dickinsii*	Fragarioides	–	Cult. Royal Botanic Garden Edinburgh 2002-0674; BROWP 1237 (E)	–	MT112953	–	MN346826	MT134227	MT134134
*Potentilla dickinsii*	Fragarioides	2*x*^a^	Crompton, D’Arcy & Coke 139 (E)	MT134118	MT112954	MT134193	MN346827	MT134228	MT134135
*Potentilla dickinsii*	Fragarioides	2*x*^a^	Sun 1989 (S)	–	MT112955	–	–	–	–
*Potentilla fragarioides*	Fragarioides	2*x*^a^	Cult. Bonn University Botanic Gardens 32074 (BONN)	MT134120	MT112957	MT134196(B) MT134197(H)	MN346828(A) MN346829(B)	MT134232(A) MT134233(D)	MT134137
*Potentilla erecta*	Reptans	4*x*^a,b^	Eriksson 1060 (BG)	MT134119	MT112956	MT134194(B) MT134195(C)	MT134154(3) MT134155(4) MT134156(16)	MT134229(2) MT134230(3) MT134231(9)	MT134136
*Potentilla indica*	Reptans	10*x*, 12*x*^a,b^	Cult. Bergen Museum Garden; Eriksson 1092 (BG)	–	MT112960	–	MT134157(I-7)	MT134238(I-1) MT134239(I-9) MT134240(I-11)	MT134140
*Potentilla indica*	Reptans	10*x*, 12*x*^a,b^	Eriksson 1061 (BG)	MT134124	MT112961	MT134205(II-W) MT134221(II-Bb) MT134206(II-Cb) MT134207(II-Kb) MT134222(T) MT134223(Y)	MT134158(II-2) MT134159(II-13) MT134160(II-19) MT134161(II-20) MT134162(II-23)	MT134241(II-3) MT134242(II-5) MT134243(II-8)	MT134141
*Potentilla reptans*	Reptans	4*x*^a,b^	Salvesen 16.45 (BG)	MT134128	MT112967	MT134215(G) MT134216(H) MT134217(I) MT134218(K)	MN346885(5) MT134175(1)	MT134254(11) MT134255(12) MT134256(823)	MT134146
*Potentilla simplex*	Reptans	–	Eriksson 797 (S)	MT134129	MT112968	MT134219(J) MT134220(P)	MT134176(2) MT134177(4) MT134178(10) MT134179(11)	MT134257(5) MT134258(7) MT134259(10) MT134260(11)	–
*Potentilla biflora*	Alba	2*x*^a^	Viereck 5042 (S)	MT134115	MT112951	–	MT134150	MT134224	MT134132
*Potentilla biflora*	Alba	2*x*^a^	Gabrielsen & Jørgensen (O)	–	–	MT134185	–	–	–
*Potentilla micrantha*	Alba	2*x*^a,b^	Cult. The Linnaean Gardens of Uppsala 1972-1035; Kårehed 432 (UPS)	MT134126	MT112964	MT134212	MT134168	MT134250	MT134143
*Potentilla sterilis*	Alba	4*x*^a,b^	Eriksson 734 (S)	MT134130	MT112969	–	MT134180(2) MT134181(7)	MT134261(3) MT134262(7)	MT134147
*Argentina anserina*	Anserina (outgroup)	4*x*, 5*x*, 6*x*^a,b^	H. Andersen BG/S-165236 (BG)	MT134115	MT112950	MT134182(C) MT134183(G) MT134184(J)	MT134149(6) MT134148(9)	–	MT134131
*Tylosperma lignosa*	Anserina (outgroup)	–	Mats Andersson 132 (GB)	MT134125	MT112963	MT134210(F) MT134211(J)	MT134166(5) MT134167(11)	MT134247(1) MT134248(6) MT134249(12)	–

### DNA extraction

DNA was extracted from 20 mg of dried leaves using the DNeasy Plant Mini Kit (Qiagen). In order to increase the amount of extracted DNA, the samples were left to lyse at 59 °C overnight before increasing the temperature to 65 °C.

### Genetic markers and DNA amplification

One chloroplast and five nuclear markers were analysed in this study; the chloroplast gene maturase K (matK), the nuclear ribosomal internal transcribed spacer (ITS) and the LCN genes dehydroascorbate reductase 2 (DHAR2), glyceraldehyde-3-phosphate dehydrogenase (GAPCP1), granule-bound starch synthase I-2 (GBSSI-2) and starch-branching enzyme I (SbeI). The forward and reverse strands of the genomes of *Fragaria vesca* (Shulaev *et al.* 2011) and *P. micrantha* (Buti *et al.* 2018) were searched through for the LCN primer sequences **[see****Supporting Information—Table S1****]**. Primer specificity was assessed by using the Search for Motifs option in Geneious version 10.2.3 (Markowitz *et al.* 2012), allowing for up to three mismatches.

DNA was amplified in a mixture of 1–20 ng total DNA, 1× Ex Taq Buffer, 0.2 mM of each dNTP, 0.4 µM of each primer, 0.75 U TaKaRa Ex Taq Hot Start Version and dH_2_O to a total volume of 25 µL. The PCR thermal cycling was run on a C1000 Touch thermal cycler (Bio-Rad Laboratories). Amplification of matK, ITS, GAPCP1, GBSSI-2 and SbeI was performed using a touchdown PCR procedure, starting with a 3 min initial denaturation at 94 °C. Then, 11 cycles of 45 s denaturation at 94 °C, 30 s of successively decreasing annealing temperatures starting at 55 °C with 0.5 °C decrement per cycle and 1 min extension at 72 °C. This was followed by 36 cycles of 45 s denaturation at 94 °C, 30 s annealing at 49 °C and 1 min extension at 72 °C, and a 7 min final extension at 72 °C. Amplification of DHAR2 was performed at higher annealing temperatures, starting with a 3 min initial denaturation at 94 °C. Then, 16 cycles of 45 s denaturation at 94 °C, 30 s of successively decreasing annealing temperatures starting at 65 °C with 0.5 °C decrement per cycle and 1 min extension at 72 °C. This was followed by 31 cycles of 45 s denaturation at 94 °C, 30 s annealing at 55 °C and 1 min extension at 72 °C, and a 7 min final extension at 72 °C. The primers used for the different markers are given in **Supporting Information—Table S1**.

### Cloning

The amplified fragments of matK and ITS displayed no or little intra-species variation and did not need cloning. This was also true for the LCN marker SbeI, and since the other three LCN markers did not show any indications of hybridization between the major clades (see Bayesian inference section), SbeI was not cloned.

PCR products from DHAR2, GAPCP1 and GBSSI-2 of species known to be polyploid or failing direct sequencing were cloned using the StrataClone PCR Cloning Kit (Agilent) following the manufacturer’s instructions. Cloned DNA was amplified in a second PCR in the same mixture as described above, only replacing DNA extract with transformed cells. The universal primers M13 forward and M13 reverse were used to amplify the cloning vector, with a 10 min initial denaturation at 94 °C, 35 cycles of 45 s denaturation at 94 °C, 45 s annealing at 55 °C and 3 min extension at 72 °C, and a 10 min final extension at 72 °C.

### Purification and sequencing

All PCR products were purified using Exosap-IT (GE Healthcare), following the manufacturer’s instructions. The number of clones sequenced for each specimen was at least 6 for tetraploids, 11 for hexaploids and 21 for decaploids, corresponding to 95 % probability of finding all gene copies (Lundberg *et al.* manuscript). The amplification primers were also used for sequencing. Sequencing reactions were performed using the BigDye Terminator Cycle Sequencing kit (Applied Biosystems) according to the manufacturer’s instructions. DNA was sequenced using an ABI Prism 3730XL DNA analyser (Applied Biosystems). All labwork was performed in the Biodiversity Lab and Sequencing Lab at the University of Bergen, Norway.

### Sequence treatments

The Staden Package (Staden 1996) and AliView v. 1.18 (Larsson 2014) were used for sequence proof reading, assembly and alignment. Scoring of uncertain or polymorphic sites was done with standard IUPAC codes. All sequences were first aligned automatically using MUSCLE (Edgar 2004), followed by manual adjustments. To identify PCR-induced inter-homoeolog recombinants (Marcussen *et al.* 2015), the sequences of cloned specimens were analysed in SplitsTree v. 4.14.6 (Huson and Bryant 2006). Those identified were removed from the alignments. All sequences have been submitted to GenBank (Table 1) and alignments have been submitted to Dataverse NO (https://doi.org/10.18710/XRQEKH).

### Model testing and Bayesian inference

Phylogenies for the individual markers were reconstructed by Bayesian inference (BI; Yang and Rannala 1997) with MrBayes v. 3.2.6 (Huelsenbeck and Ronquist 2001; Ronquist *et al.* 2012) using the MC^3^ algorithm (Altekar *et al.* 2004). The alignments of matK, DHAR2, GAPCP1, GBSSI-2 and SbeI were divided in up to five character sets each, corresponding to codon positions (3), introns (1) and indels (1). Boundaries for exons and introns were found by alignment with annotated *Fragaria* sequences from GenBank (Shulaev *et al.* 2011) and indels were coded according to the simple indel coding method of Simmons and Ochoterena (2000). Partitioning schemes and their models were based on the results from PartitonFinder2 (Lanfear *et al.* 2016) under the AICc criterium for models available in MrBayes. The Mk model (Lewis 2001) was used for the coded indels. Analyses were investigated for chain stationarity and accepted if the following criteria were fulfilled: the standard deviation of split frequencies was below 0.01, the chain swap was between 20 and 80 % (McGuire *et al.* 2007), there was no trend seen in the overlay plot and the Potential Scale Reduction Factor values (Gelman and Rubin 1992) had reached 1.0 for all parameters. The analyses were run for 5 million generations, every 1000th generation was sampled and burn-in was set to 25 or 30 %. Additional analyses were run using the same methods, taking a selective taxon removal approach by excluding either *P. dickinsii*, *P. fragarioides*, both *P. dickinsii* and *P. fragarioides* (of the Fragarioides clade), or the species of the Reptans clade, to test how this would affect the phylogeny. The trees were rooted on the Anserina clade, since it has been shown to be an outgroup to *Potentilla* (Eriksson *et al.* 2003; Töpel *et al.* 2011; Feng *et al.* 2017).

### Multispecies Coalescent analyses

Species phylogenies were inferred under the Multispecies Coalescent (MSC) model to account for ancestral polymorphisms and conflicts seen in the gene trees. The MSC model can take incomplete lineage sorting (ILS) into account, but not reticulations or gene duplication and loss (GDL) (Bravo *et al.* 2019). One ortholog is expected per set of chromosomes, and therefore we expected a single amplified fragment per chromosome set (if minor allelic variation is disregarded). Thus, for each species, the number of gene variants should be less or equal to their ploidy level (Table 1). There were no indications of reticulations in our gene trees, nor any indication of paralogs, since the expected number of gene variants was not exceeded in any species (see Bayesian inference section). Thus, we assumed that our sample did not violate the MSC model. The MSC analyses were run in *BEAST (Heled and Drummond 2010), as implemented in BEAST v. 1.8.0 (Drummond *et al.* 2012) using the same alignments as in the BI analyses. Two data sets were analysed, one including *P. dickinsii* and *P. fragarioides*, and one excluding them. The data sets comprised 19 and 17 species, respectively, in which *P. dickinsii* and *P. ancistrifolia* var. *dickinsii* were designated as the same species (Takeda 1911), as were *Ivesia kingii* and *Ivesia kingii* var. *eremica* (Ertter 1989). The substitution model for each marker was selected using PartitionFinder2 (Lanfear *et al.* 2016) under the AICc criterium for models available in BEAST. For each data set, two clock models were tested; strict and relaxed uncorrelated log normal (Drummond *et al.* 2006). For each clock model, two tree priors were tested; a birth-death process (Kendall 1948) and a birth process (Yule 1924). The analyses were run for 150 million generations, with sampling from the chain every 1000th generation, and rooted on the Anserina clade. To test the fit of the models to the data, path sampling and stepping-stone sampling (Baele *et al.* 2012, 2013) were performed with 150 steps with a length of 1 million iterations each. Log marginal likelihood differences larger than three were considered significant (Kass and Raftery 1995). Two independent analyses were run using the best-fitting models, and the results were inspected using Tracer v. 1.7.1 (Rambaut *et al.* 2018). To test that the prior did not have stronger influence over the results than the data, an additional run with sampling from prior only was performed. The tree files from the independent runs of each data set were combined using LogCombiner of the BEAST package with a burn-in of 25 % of each run. PartitionFinder2, MrBayes and BEAST were run at the CIPRES Science Gateway (Miller *et al.* 2010).

## Results

### Genetic markers

The search for the primer sites in the published genomes of *F. vesca* (Shulaev *et al.* 2011) and *Potentilla micrantha* (Buti *et al.* 2018) generated only one hit in each genome for DHAR2, GAPCP1, GBSSI-2 and SbeI, confirming their specificity.

### Bayesian inference

Models and partitioning schemes for the BI analyses are found in **Supporting Information—Table S2**. Supported clades are defined as having a posterior probability (pp) of ≥0.95.

**Figure 1.**
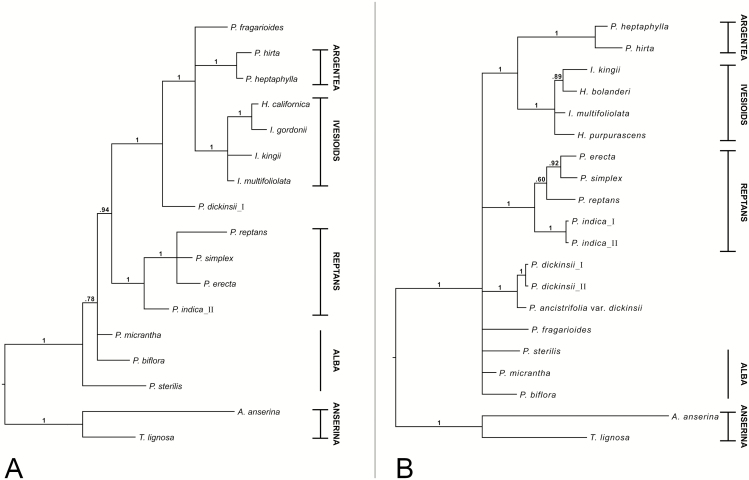
Fifty per cent majority rule consensus tree from the BI analyses of the chloroplast matK gene (A) and nuclear ribosomal ITS (B). Posterior probabilities are shown on the branch above the corresponding nodes. Specific individuals are indicated by Roman numerals. Clade affiliations of species are given to the right, where horizontal lines indicate that the clade is supported (cf. Table 1).

The matK tree with all species included (Fig. 1A) recovers the Argentea, Ivesioid and Reptans clades (all pp 1.0). The Alba species are in unresolved positions to the rest of the ingroup (pp 0.94), in which the Reptans clade is sister to a clade (pp 1.0) that consists of *P. dickinsii*, *P. fragarioides*, the Argentea clade and the Ivesioid clade. *Potentilla fragarioides*, Argentea and the Ivesioids are in a trichotomy (pp 1.0). Excluding only *P. dickinsii***[see****Supporting Information—Fig. S1****]** reduces the posterior probability for the clade of Reptans, *P. fragarioides*, Argentea and the Ivesioids from 0.94 to 0.51. When *P. fragarioides* is excluded **[see****Supporting Information—Fig. S2****]**, there are only small changes in the posterior probabilities of the tree, and the same is true in the tree in which the Reptans clade is excluded **[see****Supporting Information—Fig. S4****]**. Exclusion of both *P. fragarioides* and *P. dickinsii***[see****Supporting Information—Fig. S3****]** collapses the clade of Argentea, the Ivesioids and Reptans.

The ITS tree with all species included (Fig. 1B) recovers the Argentea, Ivesioid and Reptans clades (all pp 1.0). Apart from the Argentea and Ivesioid clades being sisters (pp 1.0), there is no other supported resolution among the clades. *Potentilla dickinsii* and *P. fragarioides* are, however, associated with the Alba species in all trees resulting from the removal analyses **[see****Supporting Information—Figs S5–S8****]**. This connection is weakly supported, except when the Reptans clade is removed **[see****Supporting Information—Fig. S8****]**. In that tree, the Alba species are in a clade (pp 1.0) with both *P. dickinsii* and *P. fragarioides* nested inside.

**Figure 2.**
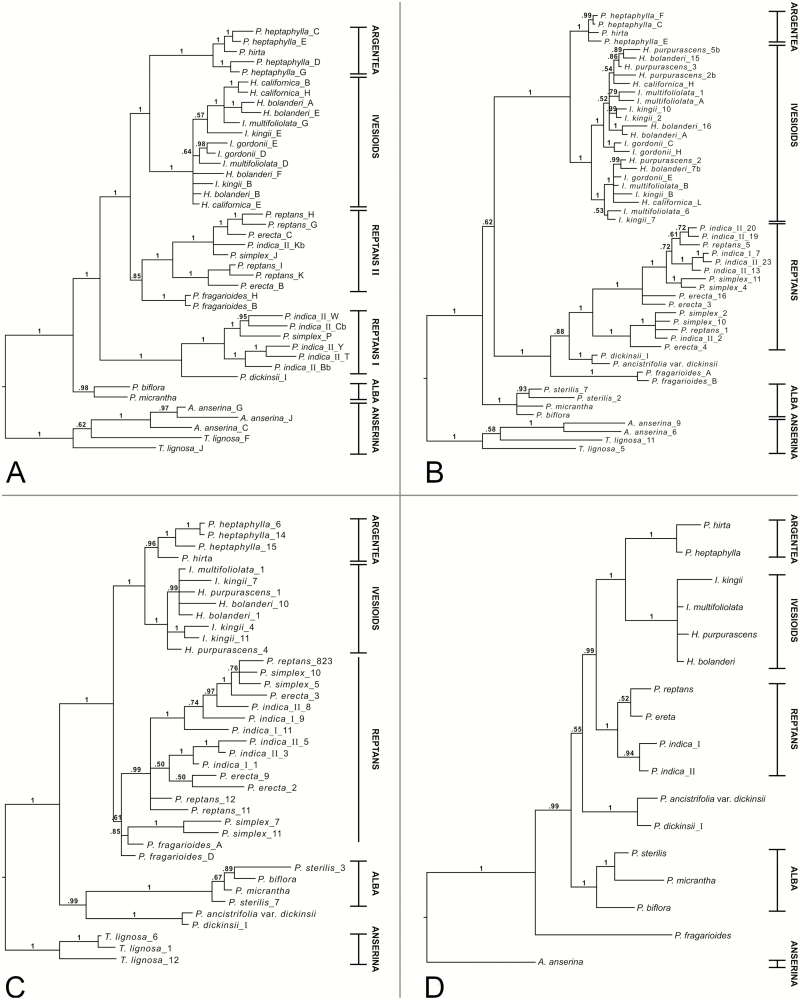
Fifty per cent majority rule consensus tree from the BI analyses of the nuclear low-copy genes DHAR2 (A), GAPCP1 (B), GBSSI-2 (C) and SbeI (D). Posterior probabilities are shown on the branch above the corresponding nodes. Specific individuals are indicated by Roman numerals. Clade affiliations of species are given within vertical lines to the right, where horizontal lines indicate that the clade is supported (cf. Table 1).

The DHAR2 tree with all species included (Fig. 2A) recovers the Argentea, Ivesioid and Alba clades (pp 1.0, 1.0 and 0.98, respectively), as well as a clade comprising Argentea and the Ivesioids (pp 1.0). In this tree, the Reptans species are divided into two clades where one (‘Reptans I’; pp 1.0) is sister (pp 1.0) to *P. dickinsii*, and the other (‘Reptans II’; pp 1.0) is sister to *P. fragarioides* with low support (pp 0.85). The clade of Reptans I plus *P. dickinsii* is sister (pp 1.0) to a clade (pp 1.0) that consists of the Reptans II plus *P. fragarioides* clade, and the clade of Argentea and the Ivesioids. There is some evidence of duplicated patterns of relationships in the Reptans II clade (*P. reptans* and *P. erecta* are sisters in both subclades; pp 1.0), as well as in the Ivesioid clade where *Horkelia bolanderi*, *H. californica* and *Ivesia multifoliolata* constitute one subclade (pp 1.0) while the other sequences of the same species are in unresolved positions outside of this subclade. None of the removal analyses **[see****Supporting Information—Figs S9–S12****]** change the topology of the trees, and there are only small changes in the posterior probabilities of the clades.

The GAPCP1 tree with all species included (Fig. 2B) recovers the Argentea, Ivesioid, Reptans and Alba clades (all pp 1.0), as well as the clade comprising Argentea and the Ivesioids (pp 1.0). A clade including all species but the Alba clade is very weakly supported (pp 0.62). Both *P. dickinsii* and *P. fragarioides* are in a clade (pp 1.0) with the Reptans clade, but the posterior probability for *P. dickinsii* being the immediate sister to Reptans is low (pp 0.88). Within the Reptans clade there are two subclades (both pp 1.0), each including gene copies of the same species, and with *P. erecta* as sister to the rest (pp 1.0). The Ivesioid clade is also divided into two subclades (both pp 1.0) with gene copies of all included Ivesioid species in each subclade, but there is no further supported pattern. When removing *P. dickinsii* there are only small changes in the posterior probabilities in the tree **[see****Supporting Information—Fig. S13****]**, but when removing *P. fragarioides***[see****Supporting Information—Fig. S14****]** and both *P. dickinsii* and *P. fragarioides***[see****Supporting Information—Fig. S15****]**, there is support for the clade including all species but the Alba clade (pp 1.0 instead of pp 0.62 or lower). Removal of the Reptans clade does not change the topology of the tree, and shows *P. dickinsii* and *P. fragarioides* as sisters (pp 1.0) **[see****Supporting Information—Fig. S16****]**.

The GBSSI-2 tree with all species included (Fig. 2C) recovers the Argentea, Ivesioid and Alba clades (pp 0.96, 1.0 and 1.0, respectively), as well as the clade comprising Argentea and the Ivesioids (pp 1.0). *Potentilla dickinsii* is sister (pp 0.99) to the Alba clade and this clade is sister (pp 1.0) to the rest of the ingroup (pp 1.0), which contains the Reptans species, *P. fragarioides* and the Argentea plus Ivesioid clade. There is some evidence of duplicated patterns of relationships in the Reptans clade, where sequences from the four included Reptans species form one subclade (pp 1.0), while the other sequences of the same species are in unresolved positions outside of this subclade. Removal of *P. dickinsii*, *P. fragarioides* or both of them does not change the topology of the trees **[see****Supporting Information—Figs S17–S19****]**. A notable change in the analysis excluding the Reptans clade **[see****Supporting Information—Fig. S20****]** is the drop in posterior probability for the Argentea clade (from pp 0.96 to pp 0.62).

The SbeI tree with all species included (Fig. 2D) recovers the Argentea, Ivesioid, Reptans and Alba clades (all pp 1.0). The Argentea and Ivesioid clades are sisters (pp 1.0), and the Reptans clade is in turn their sister (pp 0.99). *Potentilla dickinsii* is the sister of these three clades with very low support (pp 0.55), while *P. fragarioides* is supported as sister (pp 1.0) to the rest of the ingroup (pp 0.99). The removal analyses **[see****Supporting Information—Figs S21****–****S24****]** result in no changes in the topology.

### MSC analyses

**Figure 3.**
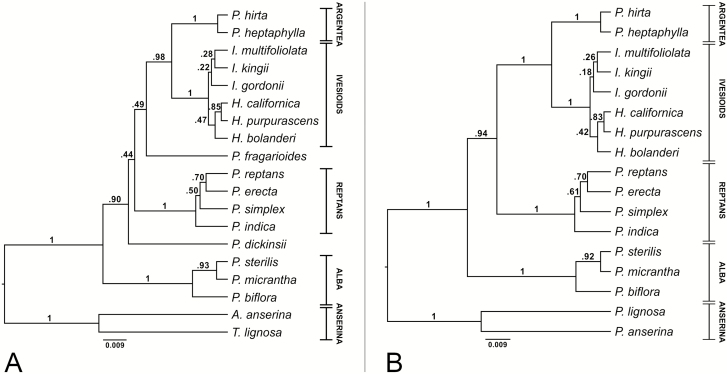
Bayesian consensus tree from the MSC analyses including *P. dickinsii* and *P. fragarioides* (A) and excluding *P. dickinsii* and *P. fragarioides* (B). Posterior probabilities are shown on the branch above the corresponding nodes. Clade affiliations of species are given within vertical lines to the right, where horizontal lines indicate that the clade is supported.

Models for the markers in the MSC analyses are found in **Supporting Information—Table S3**. For both data sets, a relaxed log-normal clock model and a birth-death process as tree prior were best fit to the data **[see****Supporting Information—Table S4****]**. The two MSC analyses recover the Argentea, Ivesioid, Reptans and Alba clades (all pp 1.0) (Fig. 3). In the analysis including *P. dickinsii* and *P. fragarioides* (Fig. 3A), the former is sister with low support (pp 0.90) to a very weakly supported clade (pp 0.44) constituting Argentea, the Ivesioids, *P. fragarioides* and Reptans, and the latter is sister with very low support (pp 0.49) to the clade (pp 0.98) of Argentea and the Ivesioids. The MSC analysis excluding *P. dickinsii* and *P. fragarioides* (Fig. 3B) shows a fully resolved tree of the major clades, where the Alba clade is sister (pp 1.0) to the rest of the ingroup (pp 0.94), in which the Reptans clade is sister to Argentea and the Ivesioids (pp 1.0).

## Discussion

This study resolves the backbone phylogeny of *Potentilla* using LCN markers. Our gene trees revealed patterns that could not have been discovered by chloroplast or nuclear ribosomal data, which makes it clear that LCN markers are crucial to the study of the evolutionary history of polyploids. Except for the Fragarioides clade, the clades found by Töpel *et al.* (2011) are supported in the majority of our gene trees.

### The Fragarioides species

In our gene trees, the Fragarioides species *P. dickinsii* and *P. fragarioides* did not constitute a clade on their own (Figs 1 and 2), except in GAPCP1 only when the Reptans species were excluded **[see****Supporting Information—Fig. S16****]**. The Fragarioides species not being resolved as a monophyletic group is in agreement with most other previous analyses, where *P. fragarioides* is resolved as sister to *P. freyniana* or *P. stolonifera* to the exclusion of *P. dickinsii* (Dobeš and Paule 2010; Töpel *et al.* 2011, chloroplast tree; Feng *et al.* 2017). The only exception seems to be in the nuclear ribosomal tree by Töpel *et al.* (2011), where *P. dickinsii* is supported as sister to *P. fragarioides* and *P. stolonifera*. We therefore suggest that *P. dickinsii* should not be treated in the same infrageneric taxon as the other Fragarioides species.

Both *P. dickinsii* and *P. fragarioides* showed several different relationships in our gene trees; *P. dickinsii* was either sister to a clade consisting of *P. fragarioides*, Argentea and the Ivesioids (matK; Fig. 1A), in an unresolved ingroup consisting of the Reptans clade, *P. fragarioides*, the Alba species and a clade with Argentea plus the Ivesioids (ITS; Fig. 1B), sister to Reptans I (DHAR2; Fig. 2A), unresolved with *P. fragarioides* and Reptans (GAPCP1; Fig. 2B), sister to Alba (GBSSI-2; Fig. 2C) or unresolved with Alba and a clade consisting of Reptans and Argentea plus the Ivesioids (SbeI; Fig. 2D). The position of *P. fragarioides* was either in an unresolved clade with Argentea and the Ivesioids (matK; Fig. 1A), in an unresolved ingroup consisting of the Reptans clade, *P. dickinsii*, the Alba species and a clade consisting of Argentea plus the Ivesioids (ITS; Fig. 1B), unresolved with Reptans II and a clade consisting of Argentea plus the Ivesioids (DHAR2; Fig. 2A), unresolved with *P. dickinsii* and Reptans (GAPCP1; Fig. 2B), unresolved with the Reptans species and Argentea plus the Ivesioids (GBSSI-2; Fig. 2C) or sister to the rest of the ingroup (SbeI; Fig. 2D). Except in a few cases, the relationships seen in the low-copy markers were not seen in our or previous chloroplast and ribosomal DNA analyses; *P. fragarioides* was sister to the rest of *Potentilla* in the ribosomal tree of Eriksson *et al.* (1998), as in our SbeI tree. In the same tree, *P. dickinsii* was sister to Alba, which is a relationship seen in our GBSSI-2 tree and in our nuclear ribosomal tree when excluding the Reptans clade **[see****Supporting Information—Figs S5****and****S8****]**.

Exclusion of one or the other of *P. dickinsii* or *P. fragarioides* did not reduce incongruence among the gene trees **[see****Supporting Information—Figs S1**, **S2**, **S5**, **S6**, **S9**, **S10**, **S13**, **S14**, **S17****and****S18****]**. However, when both *P. dickinsii* and *P. fragarioides* were excluded, the LCN markers showed the Reptans clade as sister to Argentea plus the Ivesioids (GAPCP1 and SbeI; **see****Supporting Information—Figs S15****and****S23**), or as a grade below the Argentea plus Ivesioid clade (DHAR2 and GBSSI-2; **see****Supporting Information—Figs S11****and****S19**). This topology was not contradicted by the chloroplast or ribosomal trees **[see****Supporting Information—Figs S3****and****S7**), although neither resolved these relationships with support. With this stable phylogenetic position of the Reptans clade in the backbone of the trees, we interpret *P. dickinsii* and *P. fragarioides* to be the main sources of conflicts seen in the gene phylogenies of *Potentilla*, and not the Reptans clade as initially thought.

### The Reptans clade

The Reptans clade has been monophyletic in previous phylogenetic analyses (Eriksson *et al.* 1998, 2003; Dobeš and Paule 2010; Töpel *et al.* 2011; Feng *et al.* 2017) and this was also true in most of our markers, the exceptions being DHAR2 and GBSSI-2 (Fig. 2A and C). In DHAR2, the clade was split into two clades, ‘I’ and ‘II’, where clade I was sister to *P. dickinsii* and clade II was sister with low support to *P. fragarioides*. In GBSSI-2, the clade was unresolved. The division of the Reptans clade into subclades in the DHAR2, GAPCP1 and GBSSI-2 trees (Fig. 2A–C), and all but one species being polyploid (IPCN), suggests an early genome duplication event (autopolyploidization) in this clade. This is particularly evident in the GAPCP1 tree, where there are two supported subclades, and each species is represented in both. Of the Reptans species included in our study, *P. erecta* and *P. reptans* are tetraploids, *P. indica* is deca- and dodecaploid (10*x*, 12*x*), while the ploidy level of *P. simplex* is not known (IPCN; Kurtto *et al.* 2004). We found two and three different gene variants in *P. simplex*, that were placed in different subclades, which suggests that it may also be at least tetraploid. However, it is not possible to know based on our sample if the addition of unsampled species that belong to the Reptans clade would change these patterns, and therefore additional data are required to confirm an autopolyploid origin. *Potentilla flagellaris* included in the Reptans group by Wolf (1908; in Grex Tormentillae) is reported to be diploid (Sokolovskaya *et al.* 1985), but has never been part of a phylogenetic analysis. Inclusion of this species in future analyses might shed more light on the evolutionary history of the Reptans clade.

The Reptans species *P. indica* was recently classified in the genus *Duchesnea* (Chaoluan *et al.* 2003; Ertter and Reveal 2014b; Kechaykin and Shmakov 2016), but recognition of this genus renders *Potentilla* non-monophyletic. The idea that genera, as well as other taxa, named under the International code of Botanical Nomenclature should be monophyletic is well-established in the taxonomic community (Angiosperm Phylogeny Group 1998; Backlund and Bremer 1998). All our analyses and those from previous studies (Eriksson *et al.* 1998, 2003; Töpel *et al.* 2011; Feng *et al.* 2017; Xiang *et al.* 2017) show that *P. indica* is a close relative to the type species *P. reptans*, and should therefore be included in *Potentilla*.

### The Ivesioid clade

As in the Reptans clade, the division of the Ivesioid clade into subclades in the DHAR2, GAPCP1 and GBSSI-2 trees (Fig. 2A–C), and the apparent lack of diploid species (Baldwin *et al.* 2012; IPCN), suggests an autopolyploidization event early in the clade’s history. Only a few Ivesioid species have been subject to chromosome counting, and most of them are tetraploid (4*x*) (Baldwin *et al.* 2012; IPCN). The exception is *Horkelia marinensis* (not included in this study), which is octoploid (8*x*) (Baldwin *et al.* 2012). We found between two and four gene variants in the species included in our study, but this number was not consistent across the markers, which may be indicative of extensive allele variation in addition to polyploidization.

The latest edition of Flora of North America classified the Ivesioids in the genera *Horkelia*, *Horkeliella* and *Ivesia* (Ertter and Reveal 2014a). All our analyses, as well as those from previous studies (Eriksson *et al.* 1998, 2003; Dobeš and Paule 2010; Töpel *et al.* 2011; Feng *et al.* 2017; Xiang *et al.* 2017; Zhang *et al.* 2017; Persson *et al.* 2020), consistently show that they are nested within the *Potentilla* clade. Thus, as with *Duchesnea*, recognition of these genera causes *Potentilla* to be non-monophyletic. Keeping the genera of the Ivesioid clade separate from *Potentilla* would mean that hundreds of species outside of the Reptans clade, instead of about 10 Ivesioid species, would have to be formally transferred to new genera. In addition, the recent study by Persson *et al.* (2020) suggested a history of allopolyploid speciation between the Argentea and Ivesioid clades. Such a close evolutionary relationship adds weight to the argument of the inclusion of the Ivesioid species in *Potentilla*.

### Explanations for incongruent gene trees

Given our sample and that the major clades are supported in our species trees, hybridization does not seem to have played a prominent role before they formed, but rather during their diversification. Töpel *et al.* (2011) suggested allopolyploidy as a plausible explanation for why the Reptans clade and the Fragarioides species showed different relationships in their chloroplast and ribosomal phylogenies. However, in our gene trees the Reptans species show relationships that rather indicate an autopolyploid origin of the clade (Fig. 2A–C), and *P. dickinsii* and *P. fragarioides* are diploids in all published chromosome counts (IPCN). Homoploid hybridization between diploid ancestors could explain the chromosome numbers of *P. dickinsii* and *P. fragarioides*, but both species showed several different supported relationships in the gene trees, which means that more than two parental lineages may have been involved. In that case, the incongruences cannot be explained by a single hybridization event or hybridization alone.

**Figure 4.**
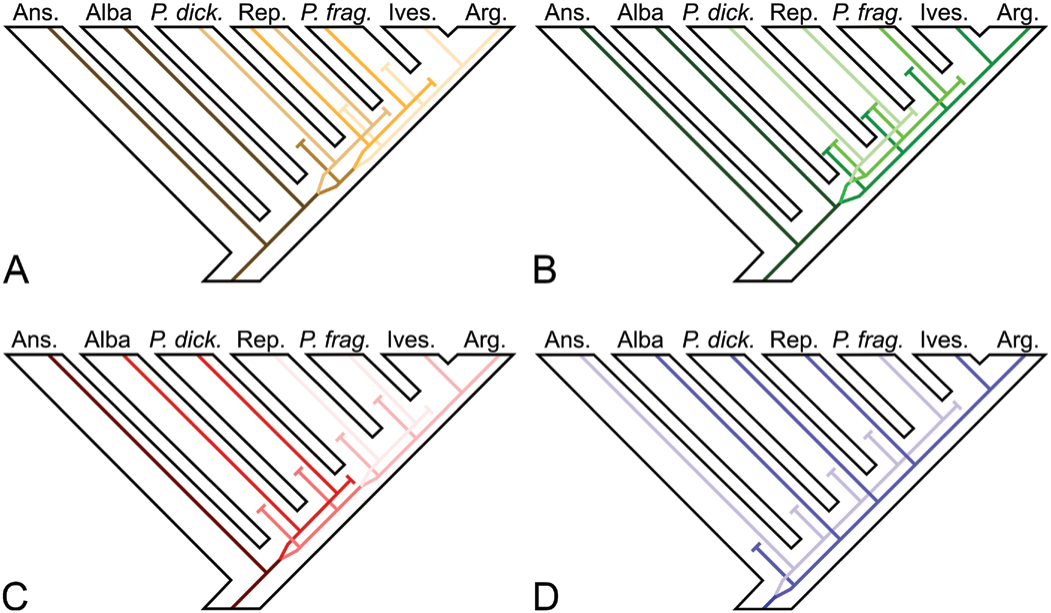
Plausible scenarios for how the gene trees of the nuclear low-copy markers in Fig. 2 may have evolved within the species phylogeny in Fig. 3 under ILS. Colours indicate different gene variants. (A) DHAR2, (B) GAPCP1, (C) GBSSI-2, (D) SbeI. Abbreviations: Ans. = Anserina clade; *P. dick.* = *P. dickinsii*; Rep. = Reptans clade; *P. frag.* = *P. fragarioides*; Ives. = Ivesioid clade; Arg. = Argentea clade.

In addition to hybridization, ILS is an evolutionary process that can lead to conflicting gene phylogenies (Doyle 1992; Maddison 1997). Gene trees usually coalesce deeper than the speciation events and are therefore expected to differ from the actual species phylogeny (Oxelman *et al.* 2017). Figure 4 shows how the LCN phylogenies in Fig. 2 may be contained within the species phylogeny in Fig. 3A. Assuming there were no polyploidizations or hybridizations between lineages before radiation of the clades, we interpret the gene variants conserved to have evolved before the time of diversification of the different clades. In DHAR2 (Fig. 4A), the Reptans species are divided into the Reptans I and II clades, where I is sister to *P. dickinsii* and II is sister (with low support) to *P. fragarioides*. Therefore, under this interpretation, a second gene variant evolved at least before the divergence of *P. dickinsii*, where one variant is conserved in the Reptans I and *P. dickinsii* lineage. The other variant evolved into two new variants before the divergence of Reptans II, and one of those variants is conserved in the Reptans II and *P. fragarioides* lineage. In GAPCP1 (Fig. 4B), *P. dickinsii* is sister to Reptans, and *P. fragarioides* is in turn their sister. Therefore, a second gene variant evolved at least before *P. dickinsii* diverged. One of those variants evolved into two new variants, where one is conserved in *P. fragarioides* and the other one in *P. dickinsii* and Reptans. In GBSSI-2 (Fig. 4C), *P. dickinsii* is sister to Alba, and therefore a second gene variant evolved at least before divergence of Alba, where one variant is conserved in these two lineages. There was very low support for *P. fragarioides* being sister to Reptans in the GBSSI-2 tree, but there might have evolved two new variants from the one variant not conserved in Alba and *P. dickinsii* before the divergence of the Reptans lineage. One of those variants was then conserved in Reptans and *P. fragarioides*. In SbeI (Fig. 4D), *P. fragarioides* is sister to the rest of the ingroup (due to rooting on the Anserina clade). Therefore, a second gene variant evolved before the Anserina lineage diverged. One of those variants is conserved in Anserina and *P. fragarioides*, and the other one in Alba, Reptans, *P. dickinsii*, the Ivesioids and Argentea. No marker is immune to ILS, but a larger number of unlinked nuclear low-copy markers applied in a MSC model could potentially resolve the relationships of *P. dickinsii* and *P. fragarioides* to the major clades of *Potentilla*.

### Species trees and the backbone phylogeny

Since there were no indications of reticulate relationships between the clades in our gene trees, we performed MSC analyses to infer species trees. This was done to see if the shared patterns in the gene trees when *P. dickinsii* and *P. fragarioides* were excluded would be confirmed. This kind of analysis is advantageous over concatenation, since the model is able to take ILS and different histories of loci into account (Degnan and Rosenberg 2009). In addition, concatenation would not be possible for the cloned markers, since we do not know which gene variants belong to the same chromosome sets. The MSC analysis excluding *P. dickinsii* and *P. fragarioides* showed a fully resolved tree down to the level of the previously defined clades (Fig. 3B); where Alba was sister to the rest of the ingroup (pp 0.94), in which Reptans was sister to Argentea plus the Ivesioids (pp 1.0). As expected, the tree was not fully resolved when *P. dickinsii* and *P. fragarioides* were included (Fig. 3A) since the nodes directly related to the position of *P. dickinsii* and *P. fragarioides* were not supported. The low resolution within the Ivesioid and Reptans clades may be due to the presumably autopolyploid origins of these clades, as indicated by our interpretation of the gene tree topologies.

Recombination and hybridization are evolutionary processes that violate the MSC model (Bravo *et al.* 2019). Those processes result in reticulate relationships, and allopolyploid species are known to occur in *Potentilla* (Paule *et al.* 2011; Persson *et al.* 2020). Due to both auto- and allopolyploid taxa being present in the genus, it is evident that the complete evolutionary history of *Potentilla*, as opposed to the backbone relationships, may only be possible to describe correctly with a reticulate tree.

### Sampling effects

It is clear from our results that inferred relationships may be strongly affected by the inclusion or exclusion of single species. In our study, we focused on the relationships between the major clades, exploring under which sampling regimes we would get a supported phylogenetic backbone for *Potentilla*. This meant that we included representatives of the most well-supported clades, but also that some groups were excluded. In particular, we did not sample species of the Himalayan clade that were previously classified in *Sibbaldia* (Eriksson *et al.* 2015). In previous analyses using chloroplast and nuclear ribosomal data (Dobeš and Paule 2010; Eriksson *et al.* 2015; Feng *et al.* 2017), this clade is either resolved as sister to Alba or in an unresolved position in relation to Alba and the rest of *Potentilla*. Thus, inclusion of this clade would have been unlikely to affect the results presented here. There are possibly other species in addition to *P. dickinsii* and *P. fragarioides* that might affect the phylogeny in similar ways, but if so, they are still to be sampled for phylogenetic analysis. Inclusion of any close relatives to *P. dickinsii* and *P. fragarioides* in future studies could potentially stabilize their positions in the tree, and reveal more information about putative hybridizations in their evolutionary history.

## Conclusions

In this study, we have found a supported phylogenetic backbone of *Potentilla*, based on the relationships between the four major clades of *Potentilla*: the Alba clade as sister to the rest, then the Reptans clade, and then the Argentea clade as sister to the Ivesioid clade.

The different nuclear low-copy genes show incongruent phylogenetic relationships in our sample of *Potentilla* species, and we conclude that these incongruences are mainly caused by *P. dickinsii* and *P. fragarioides*.

*Potentilla dickinsii* and *P. fragarioides* have sometimes been joined in the informal Fragarioides group. We have no results that support this grouping as monophyletic, and suggest that these species should not be classified in the same infrageneric taxon.

We found no evidence in our sample for any hybridization or allopolyploidization events between the major clades, and suggest that early *Potentilla* evolution was affected by other processes such as ILS.

Possible autopolyploidization events were inferred in the Reptans and Ivesioid clades.

This study adds to the abundant molecular evidence that a monophyletic status of *Potentilla* would be achieved by an inclusion of all the Ivesioid genera (*Horkelia*, *Horkeliella* and *Ivesia*), as well as *Duchesnea*.

## Supporting Information

The following additional information is available in the online version of this article—

**Table S1.** Primer pairs used for amplification of the markers analysed.

**Table S2.** Evolutionary models for the different markers in the Bayesian inference analyses.

**Table S3.** Evolutionary models for the different markers in the Multispecies Coalescent analyses.

**Table S4.** Log marginal likelihood values for analyses in *BEAST.

**Figures S1–S24.** Consensus trees from the Bayesian inference analyses.

plaa017_suppl_Supplementary-Figures_S1-S24

plaa017_suppl_Supplementary_Table_S1

plaa017_suppl_Supplementary_Table_S2

plaa017_suppl_Supplementary_Table_S3

plaa017_suppl_Supplementary_Table_S4
