# Supplementary material for: Detecting destabilizing species in the phylogenetic backbone of Potentilla (Rosaceae) using low-copy nuclear markers
Source: AoB Plants. 2020 May 9;12(3):plaa017. doi: 10.1093/aobpla/plaa017 (PMC7287270; doi:10.1093/aobpla/plaa017)
Supplement: plaa017_suppl_Supplementary-Figures_S1-S24 [file plaa017_suppl_supplementary-figures_s1-s24.pdf]

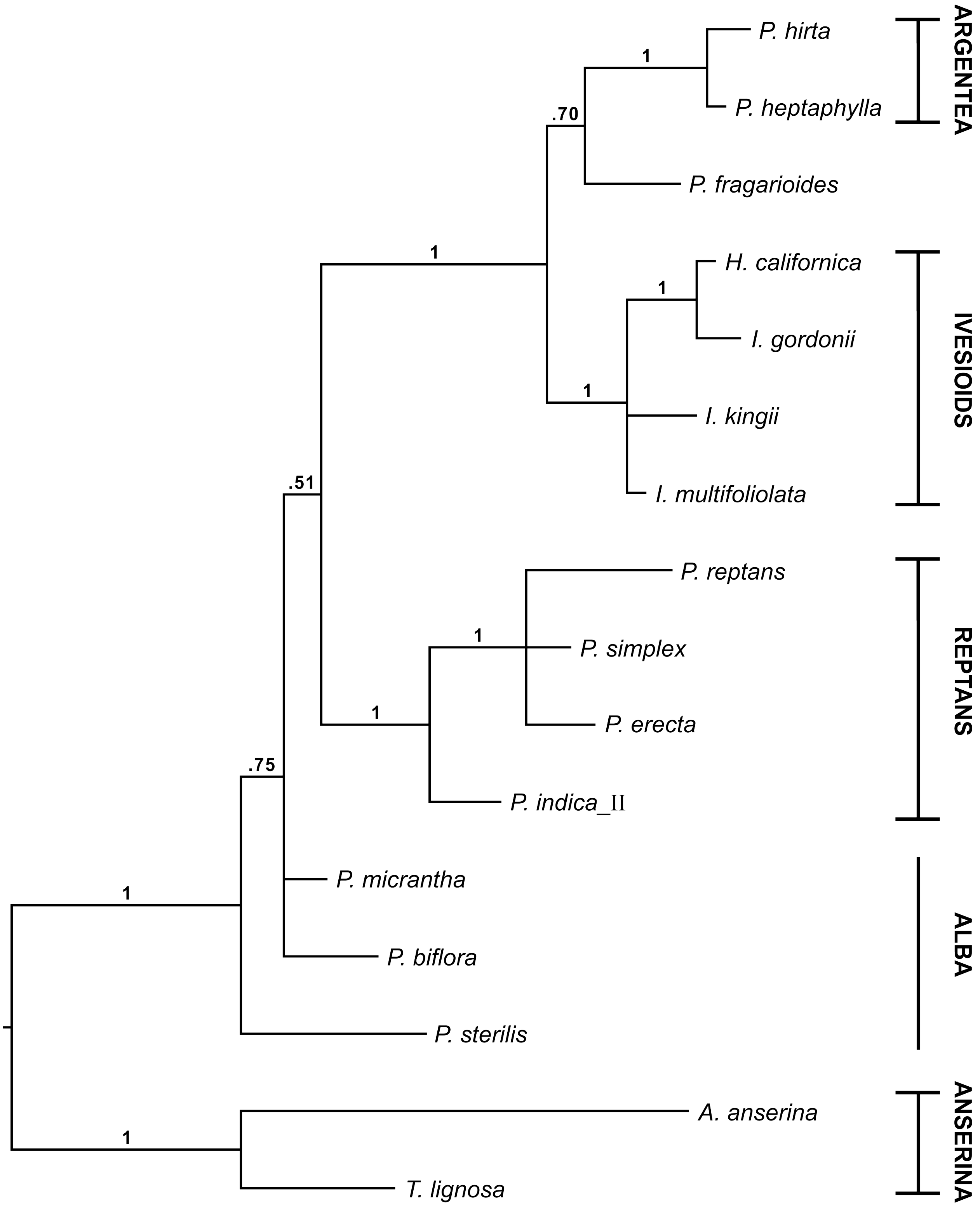

Figure S1. 50% majority rule consensus tree from the Bayesian Inference analysis of the chloroplast matK gene, excluding *P. dickinsii*. Bayesian Inference posterior probabilities are shown on the branch above the corresponding nodes. Specific individuals are indicated by Roman numerals. Clade affiliations of species are given to the right, where horizontal lines indicate that the clade is supported (cf. Table 1).

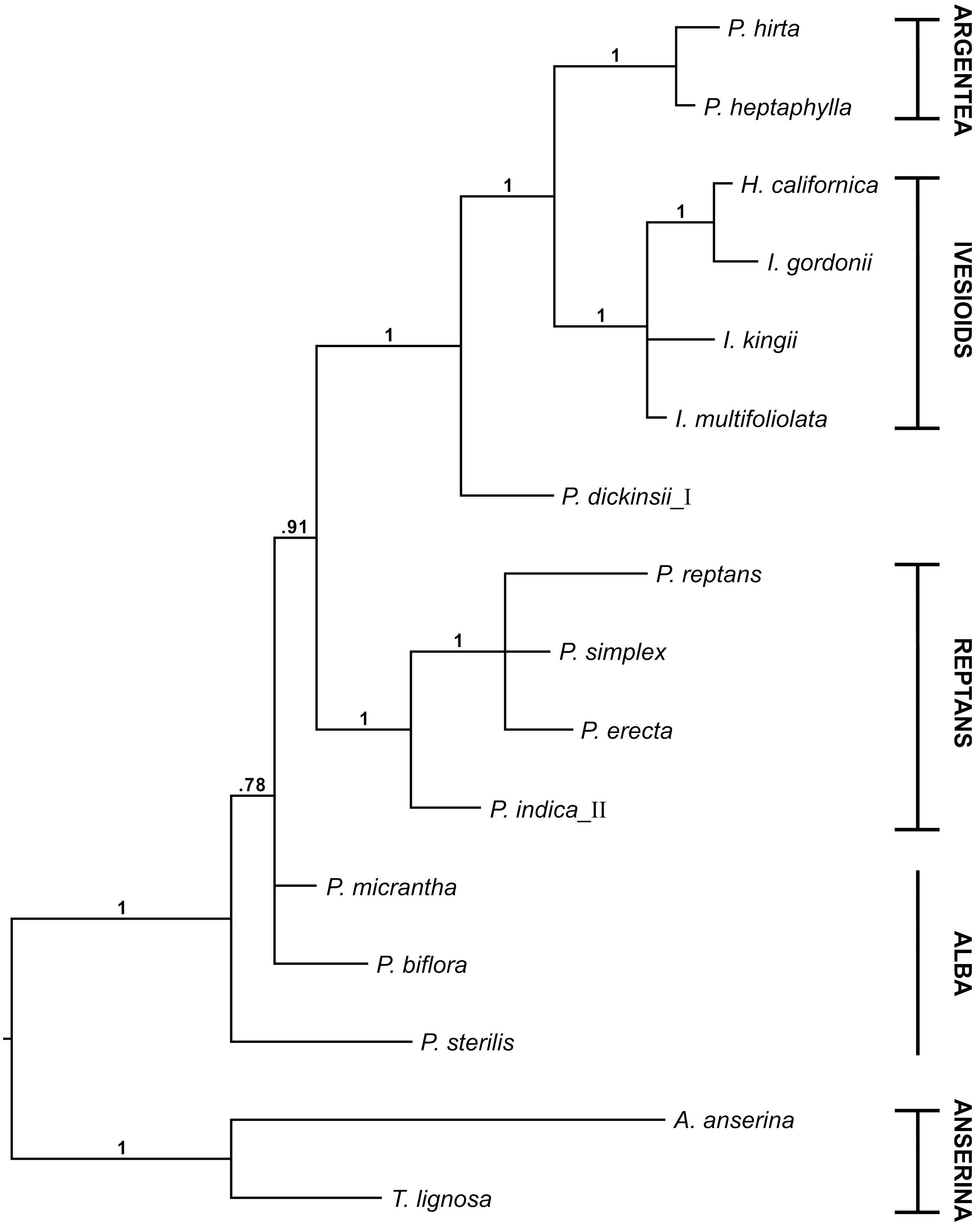

Figure S2. 50% majority rule consensus tree from the Bayesian Inference analysis of the chloroplast matK gene, excluding *P. fragarioides*. Bayesian Inference posterior probabilities are shown on the branch above the corresponding nodes. Specific individuals are indicated by Roman numerals. Clade affiliations of species are given to the right, where horizontal lines indicate that the clade is supported (cf. Table 1).

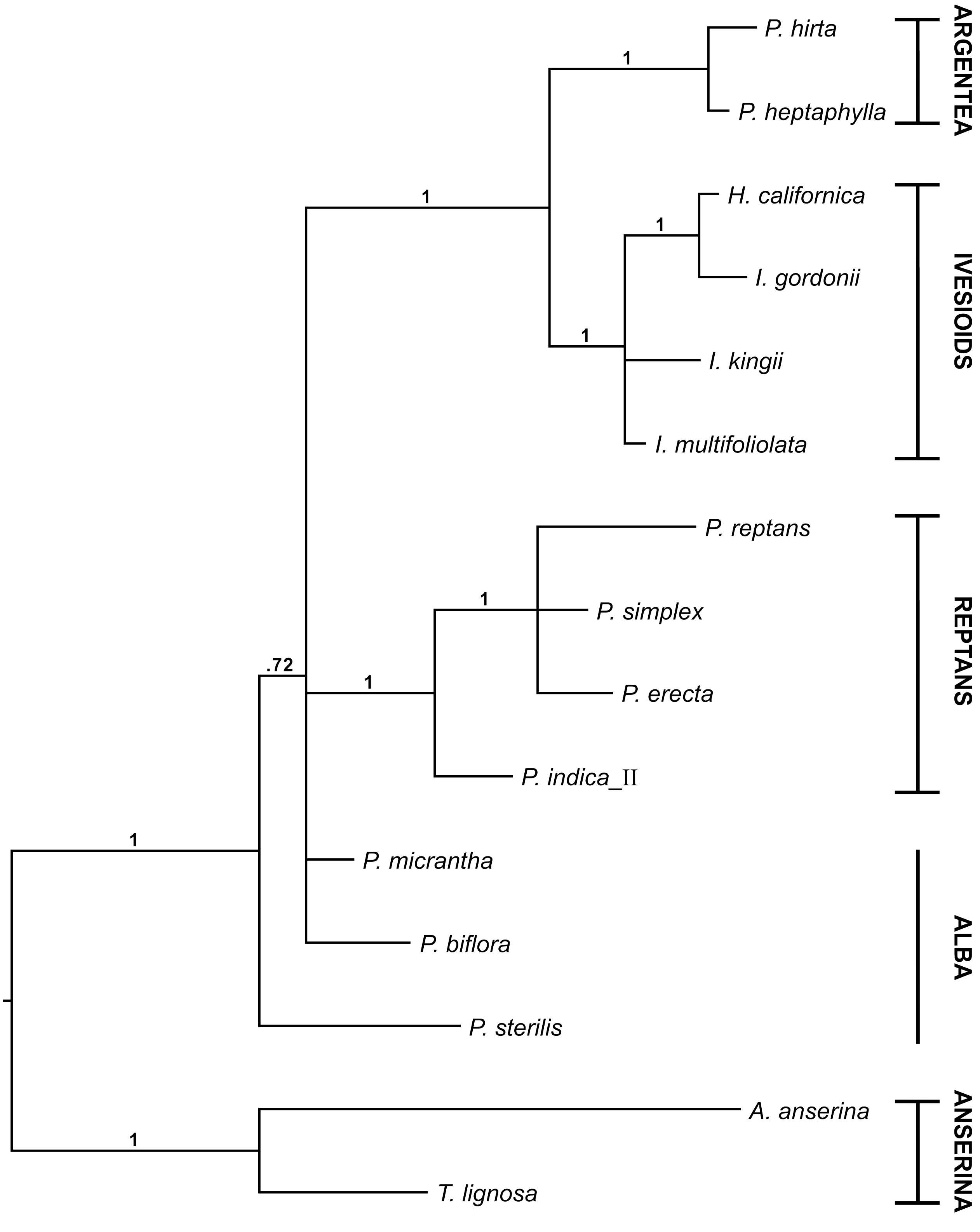

Figure S3. 50% majority rule consensus tree from the Bayesian Inference analysis of the chloroplast matK gene, excluding *P. dickinsii* and *P. fragarioides*. Bayesian Inference posterior probabilities are shown on the branch above the corresponding nodes. Specific individuals are indicated by Roman numerals. Clade affiliations of species are given to the right, where horizontal lines indicate that the clade is supported (cf. Table 1).

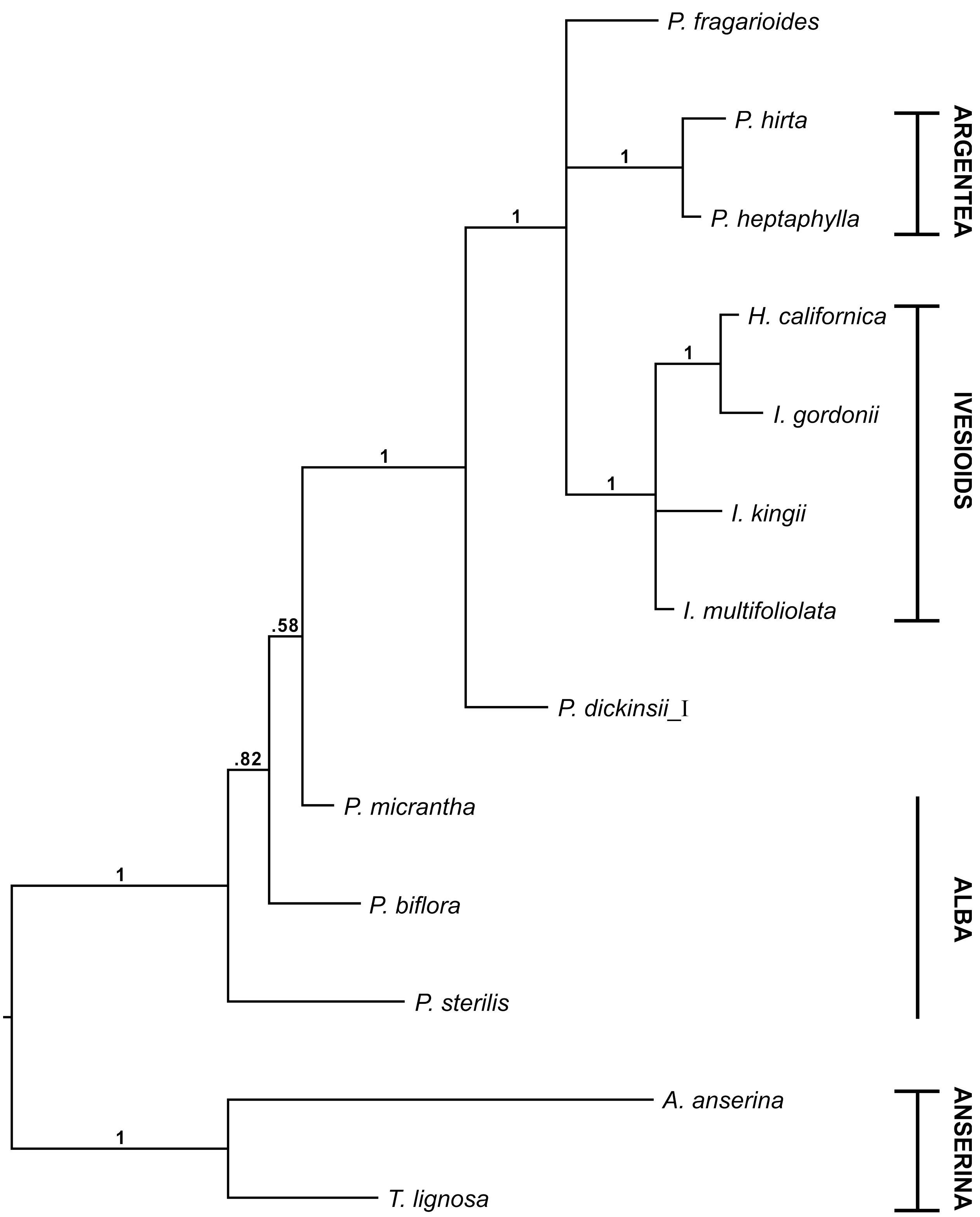

Figure S4. 50% majority rule consensus tree from the Bayesian Inference analysis of the chloroplast matK gene, excluding the Reptans clade. Bayesian Inference posterior probabilities are shown on the branch above the corresponding nodes. Specific individuals are indicated by Roman numerals. Clade affiliations of species are given to the right, where horizontal lines indicate that the clade is supported (cf. Table 1).

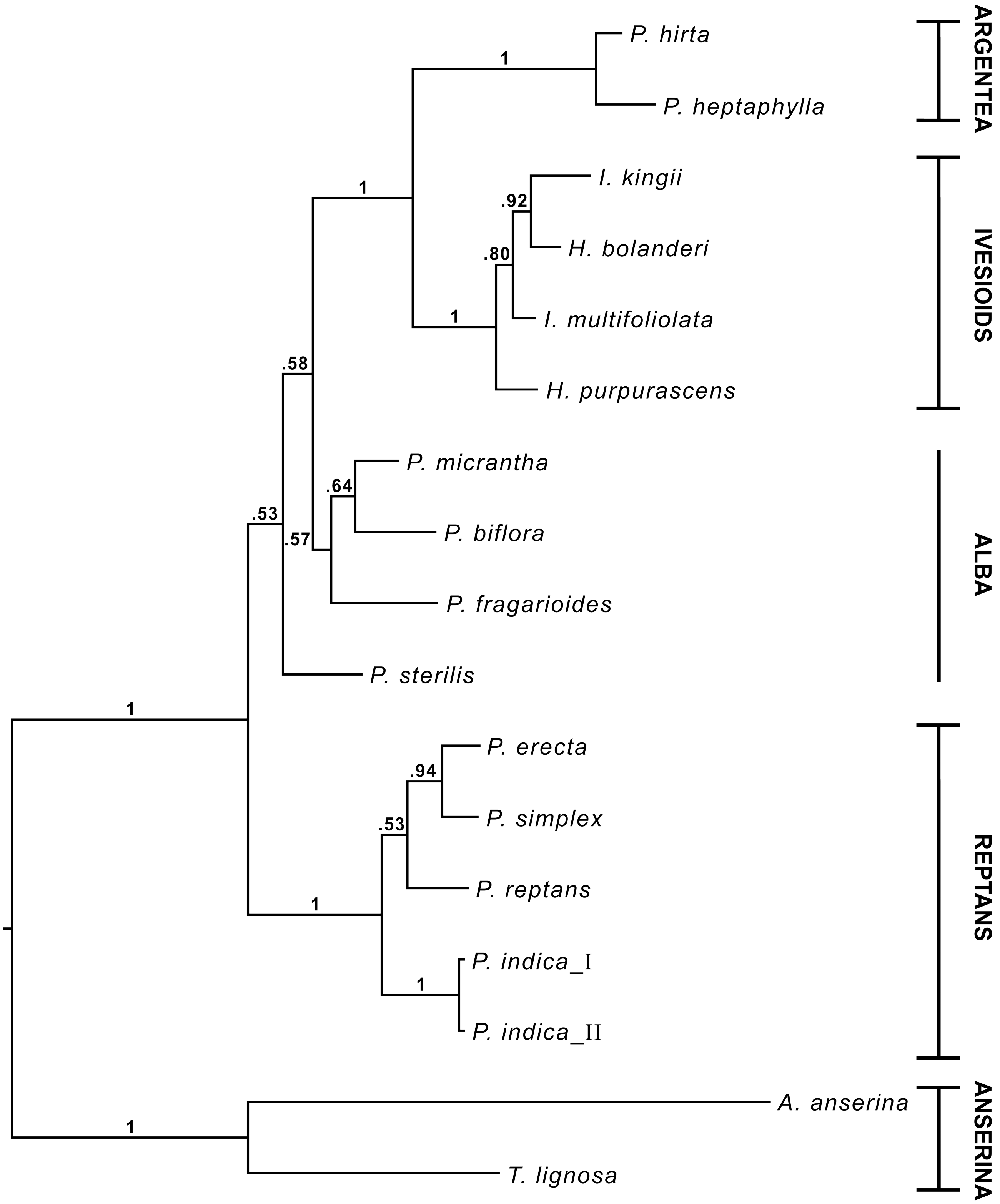

Figure S5. 50% majority rule consensus tree from the Bayesian Inference analysis of the nuclear ribosomal ITS, excluding *P. dickinsii*. Bayesian Inference posterior probabilities are shown on the branch above the corresponding nodes. Specific individuals are indicated by Roman numerals. Clade affiliations of species are given to the right, where horizontal lines indicate that the clade is supported (cf. Table 1).

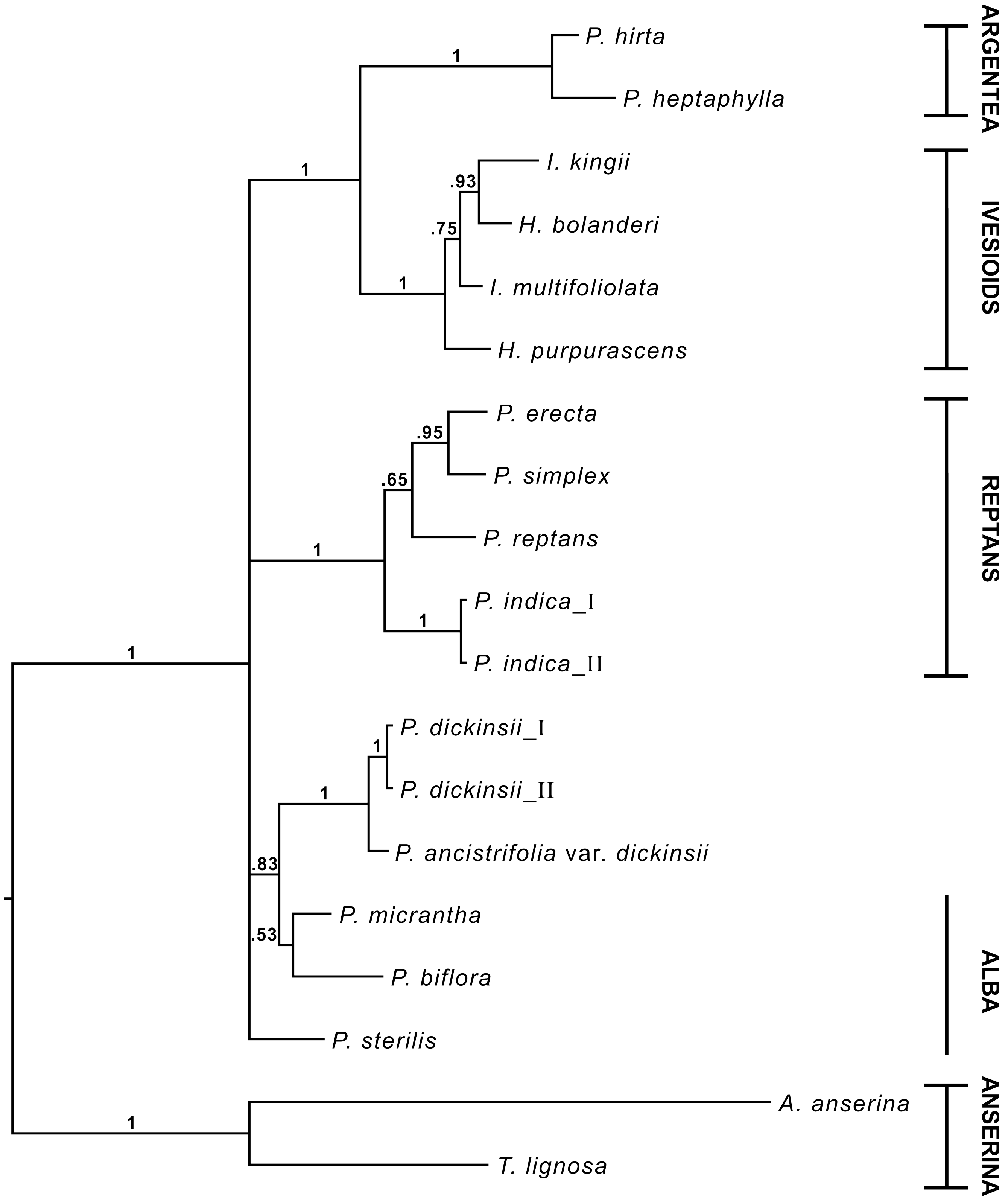

Figure S6. 50% majority rule consensus tree from the Bayesian Inference analysis of the nuclear ribosomal ITS, excluding *P. fragarioides*. Bayesian Inference posterior probabilities are shown on the branch above the corresponding nodes. Specific individuals are indicated by Roman numerals. Clade affiliations of species are given to the right, where horizontal lines indicate that the clade is supported (cf. Table 1).

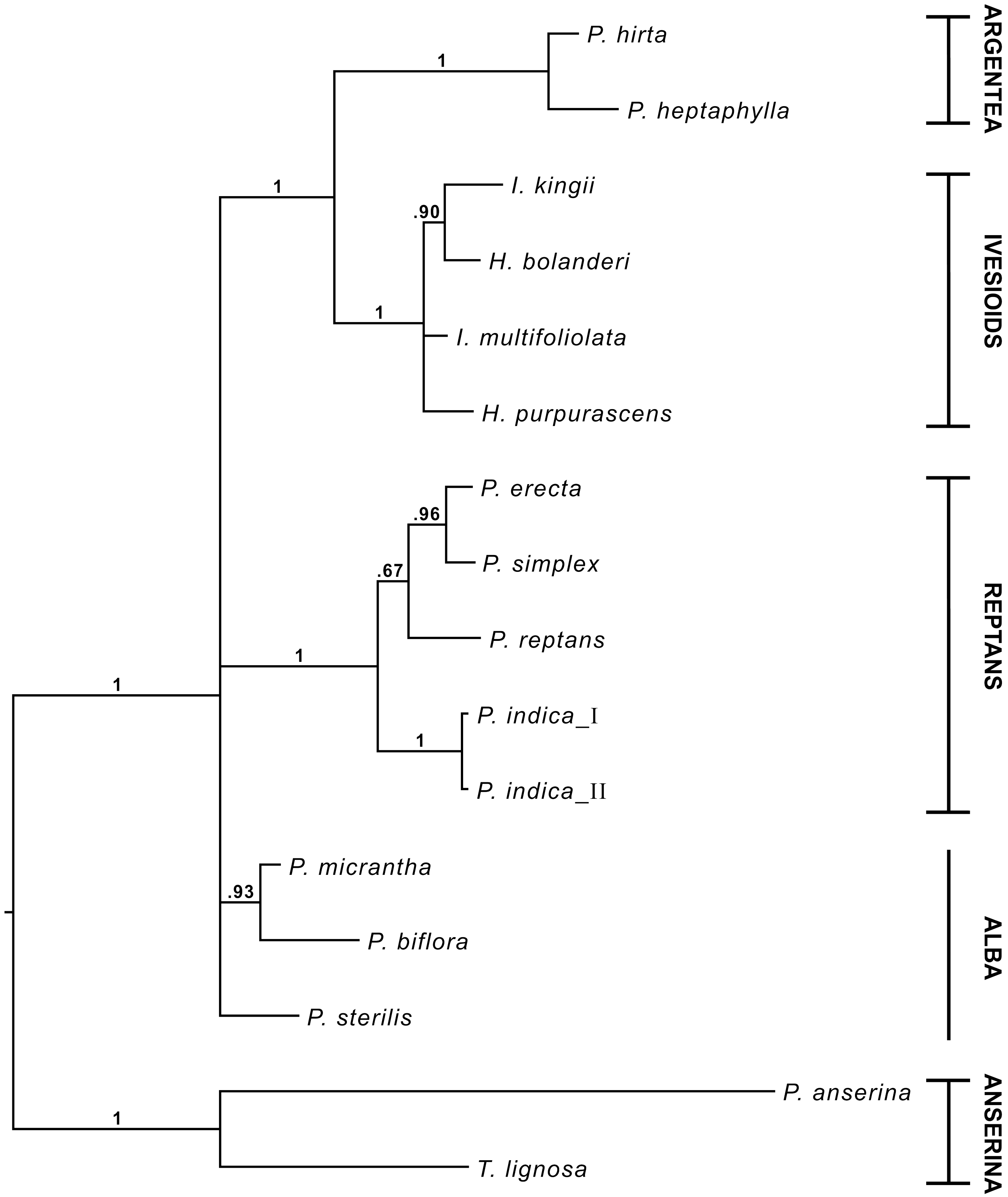

Figure S7. 50% majority rule consensus tree from the Bayesian Inference analysis of the nuclear ribosomal ITS, excluding *P. dickinsii* and *P. fragarioides*. Bayesian Inference posterior probabilities are shown on the branch above the corresponding nodes. Specific individuals are indicated by Roman numerals. Clade affiliations of species are given to the right, where horizontal lines indicate that the clade is supported (cf. Table 1).

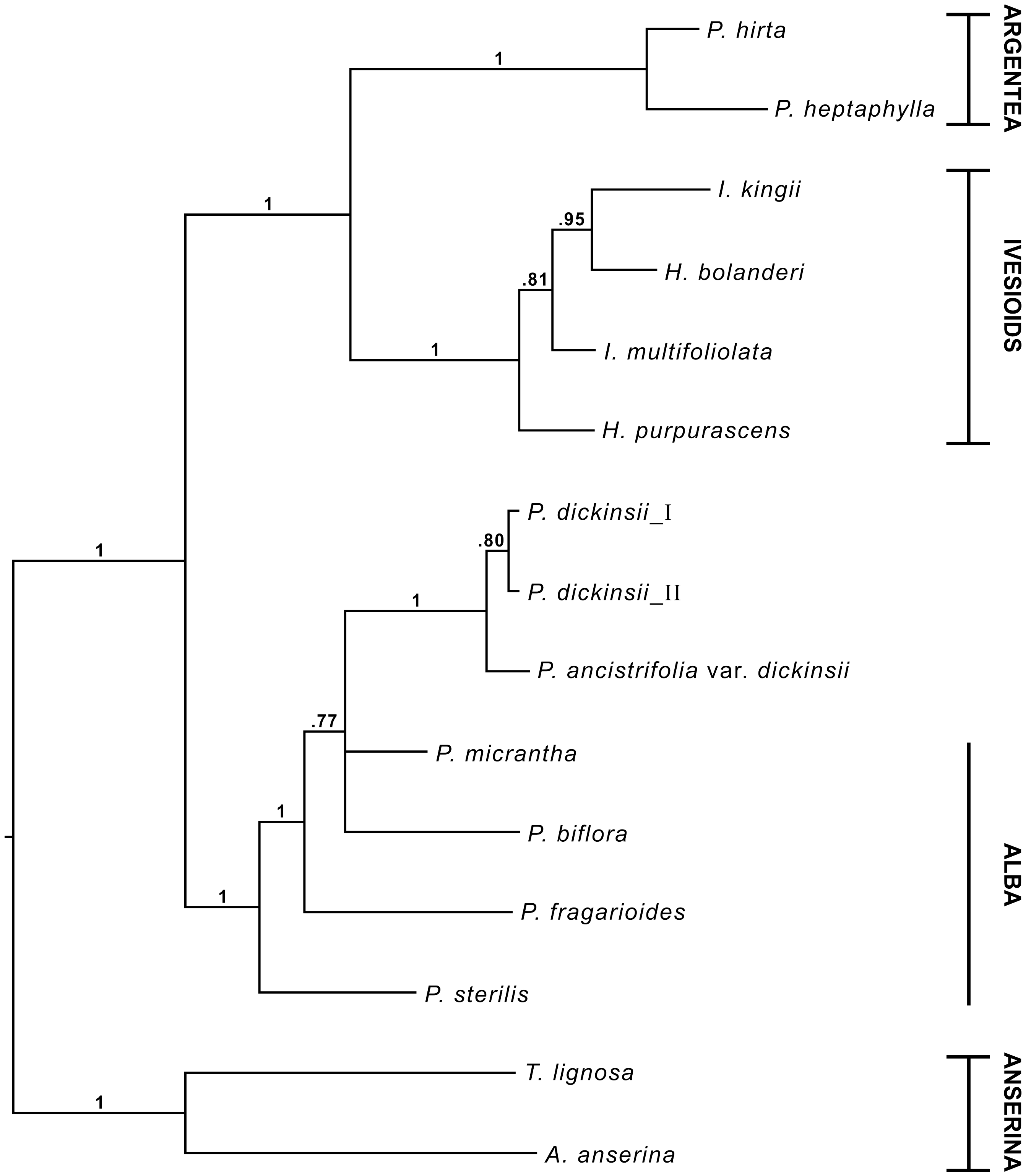

Figure S8. 50% majority rule consensus tree from the Bayesian Inference analysis of the nuclear ribosomal ITS, excluding the Reptans clade. Bayesian Inference posterior probabilities are shown on the branch above the corresponding nodes. Specific individuals are indicated by Roman numerals. Clade affiliations of species are given to the right, where horizontal lines indicate that the clade is supported (cf. Table 1).

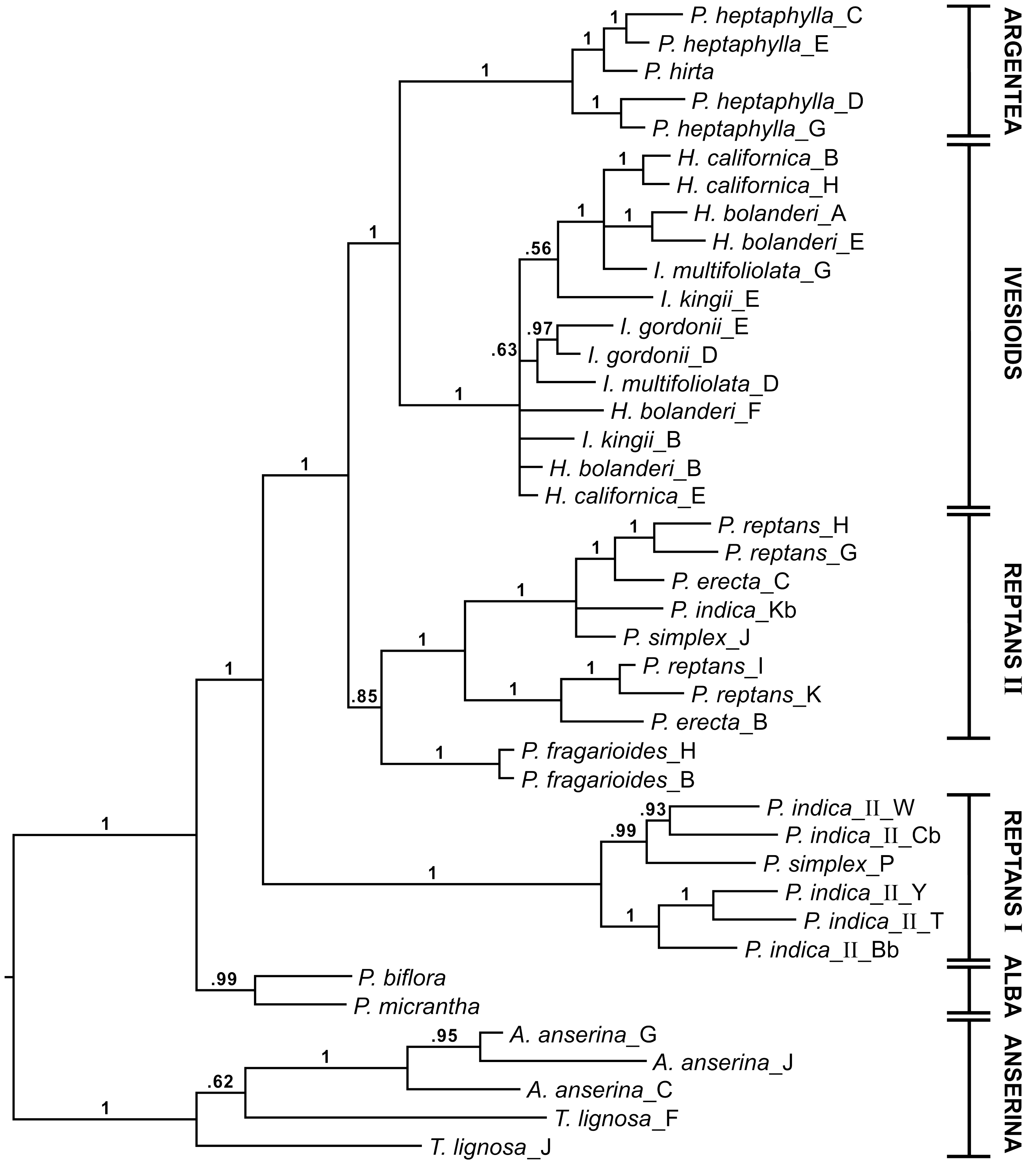

Figure S9. 50% majority rule consensus tree from the Bayesian Inference analysis of the nuclear low-copy DHAR2 gene, excluding *P. dickinsii*. Bayesian Inference posterior probabilities are shown on the branch above the corresponding nodes. Specific individuals are indicated by Roman numerals and clones are indicated by letters. Clade affiliations of species are given to the right, where horizontal lines indicate that the clade is supported (cf. Table 1).

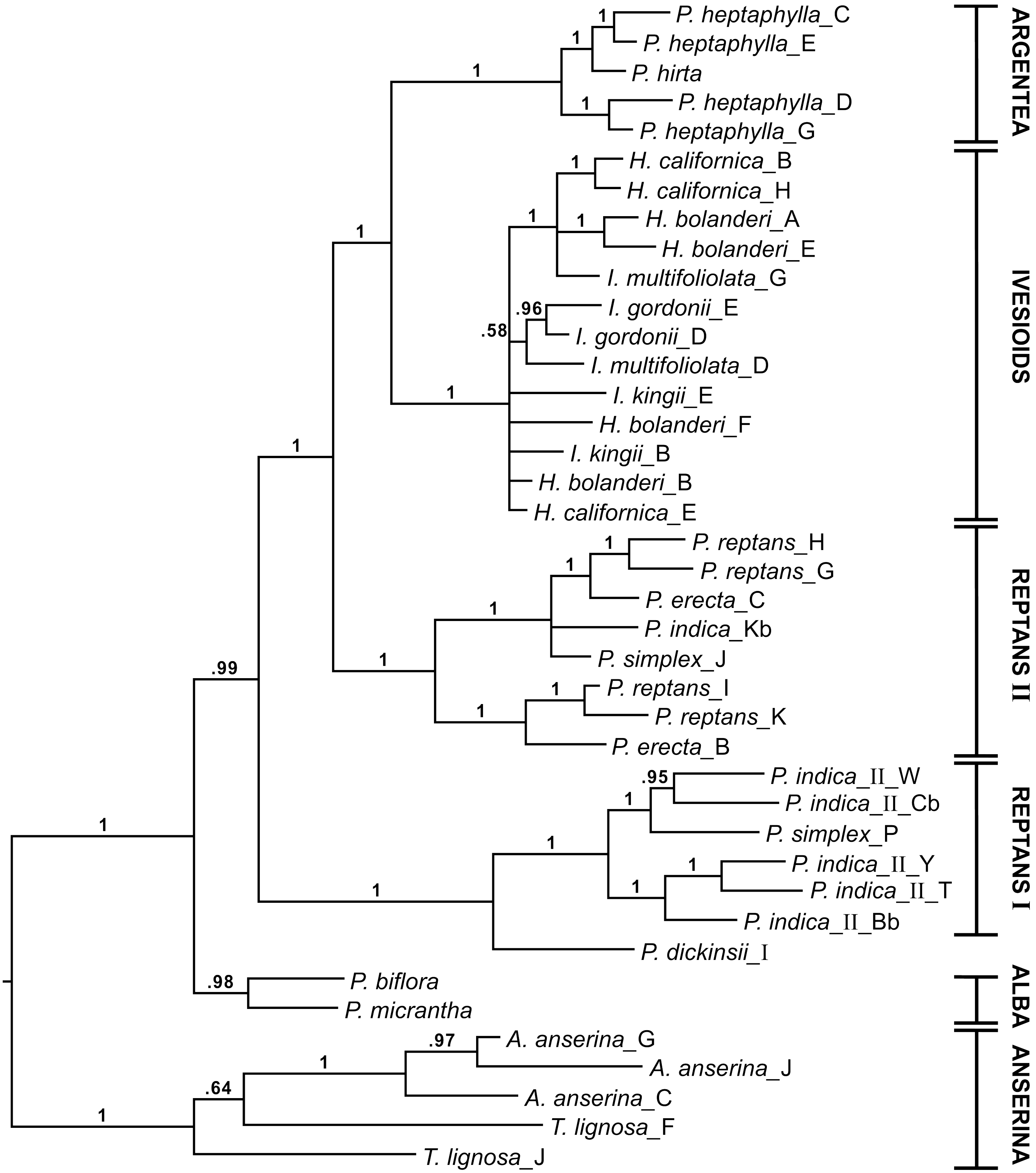

Figure S10. 50% majority rule consensus tree from the Bayesian Inference analysis of the nuclear low-copy DHAR2 gene, excluding *P. fragarioides*. Bayesian Inference posterior probabilities are shown on the branch above the corresponding nodes. Specific individuals are indicated by Roman numerals and clones are indicated by letters. Clade affiliations of species are given to the right, where horizontal lines indicate that the clade is supported (cf. Table 1).

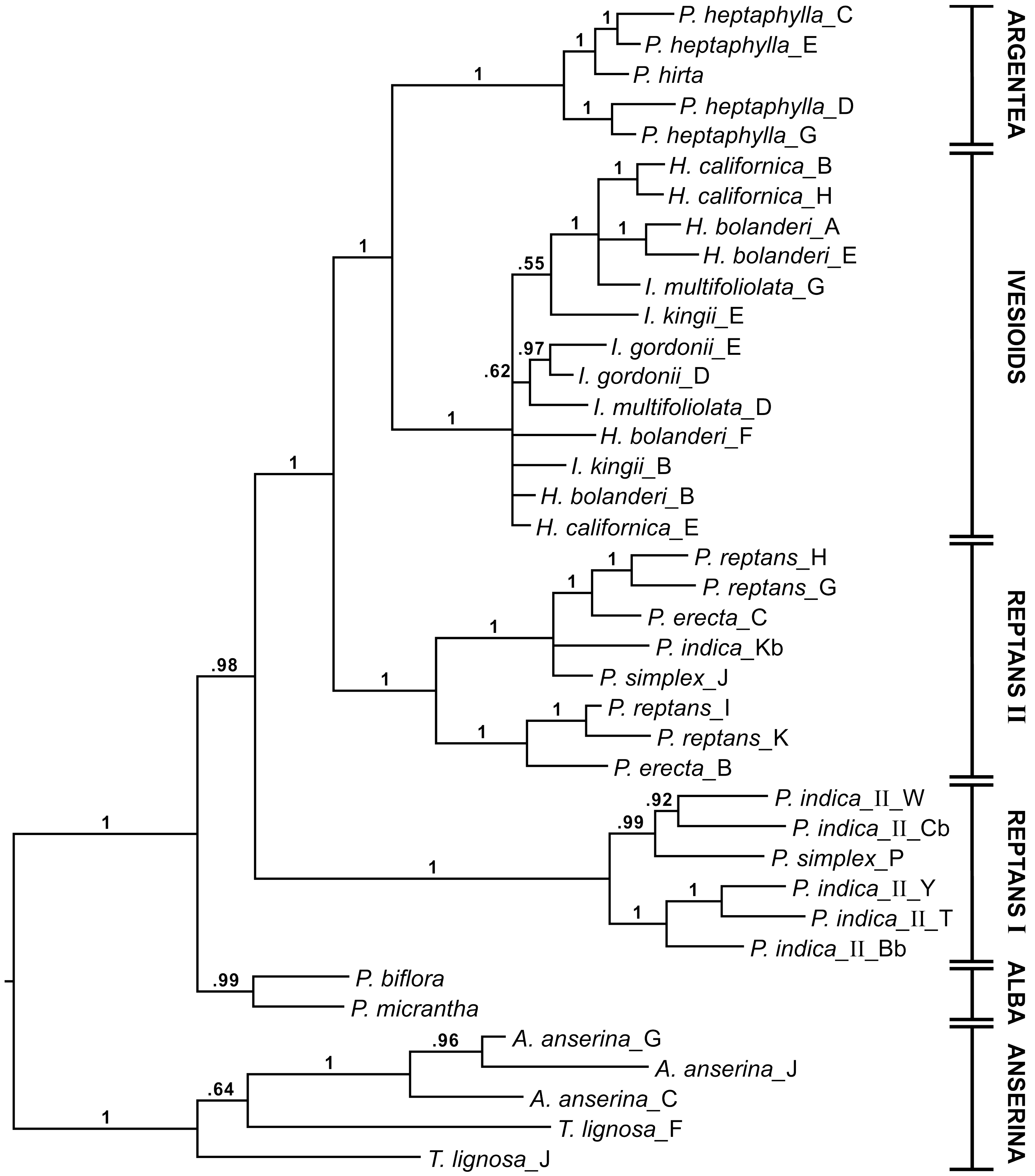

Figure S11. 50% majority rule consensus tree from the Bayesian Inference analysis of the nuclear low-copy DHAR2 gene, excluding *P. dickinsii* and *P. fragarioides*. Bayesian Inference posterior probabilities are shown on the branch above the corresponding nodes. Specific individuals are indicated by Roman numerals and clones are indicated by letters. Clade affiliations of species are given to the right, where horizontal lines indicate that the clade is supported (cf. Table 1).

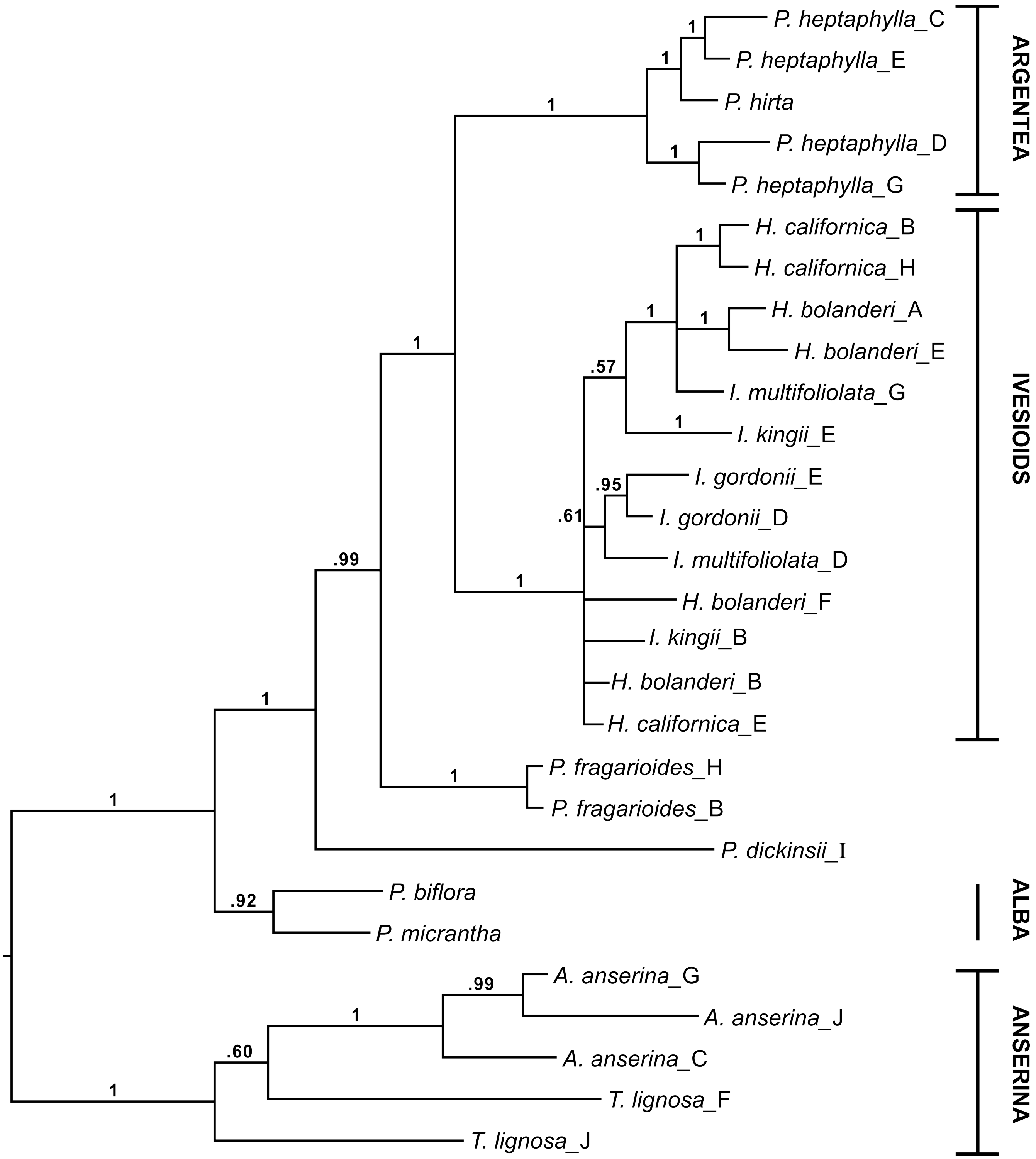

Figure S12. 50% majority rule consensus tree from the Bayesian Inference analysis of the nuclear low-copy DHAR2 gene, excluding the Reptans clade. Bayesian Inference posterior probabilities are shown on the branch above the corresponding nodes. Specific individuals are indicated by Roman numerals and clones are indicated by letters. Clade affiliations of species are given to the right, where horizontal lines indicate that the clade is supported (cf. Table 1).

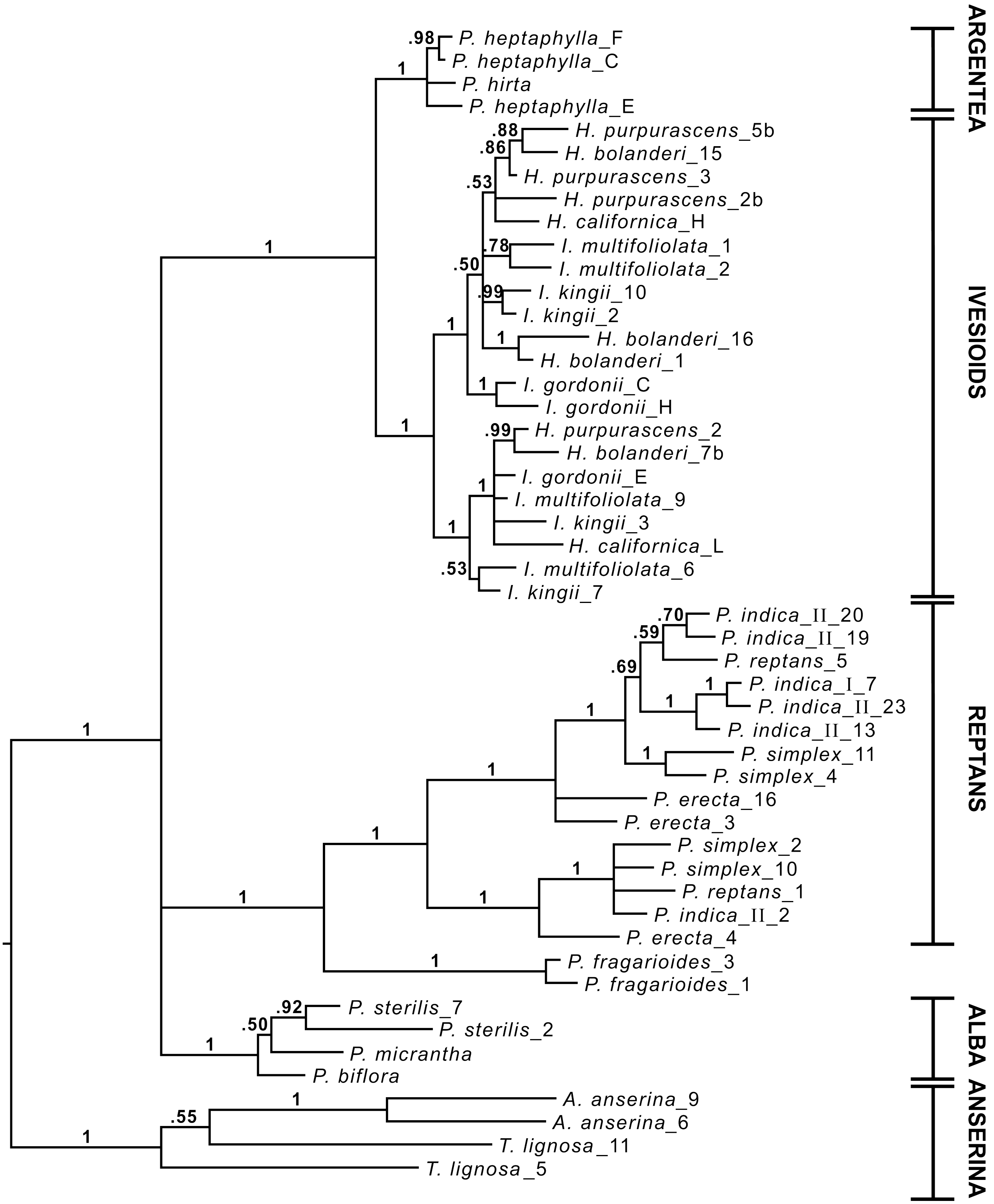

Figure S13. 50% majority rule consensus tree from the Bayesian Inference analysis of the nuclear low-copy GAPCP1 gene, excluding *P. dickinsii*. Bayesian Inference posterior probabilities are shown on the branch above the corresponding nodes. Specific individuals are indicated by Roman numerals and clones are indicated by letters and Arabic numbers. Clade affiliations of species are given to the right, where horizontal lines indicate that the clade is supported (cf. Table 1).

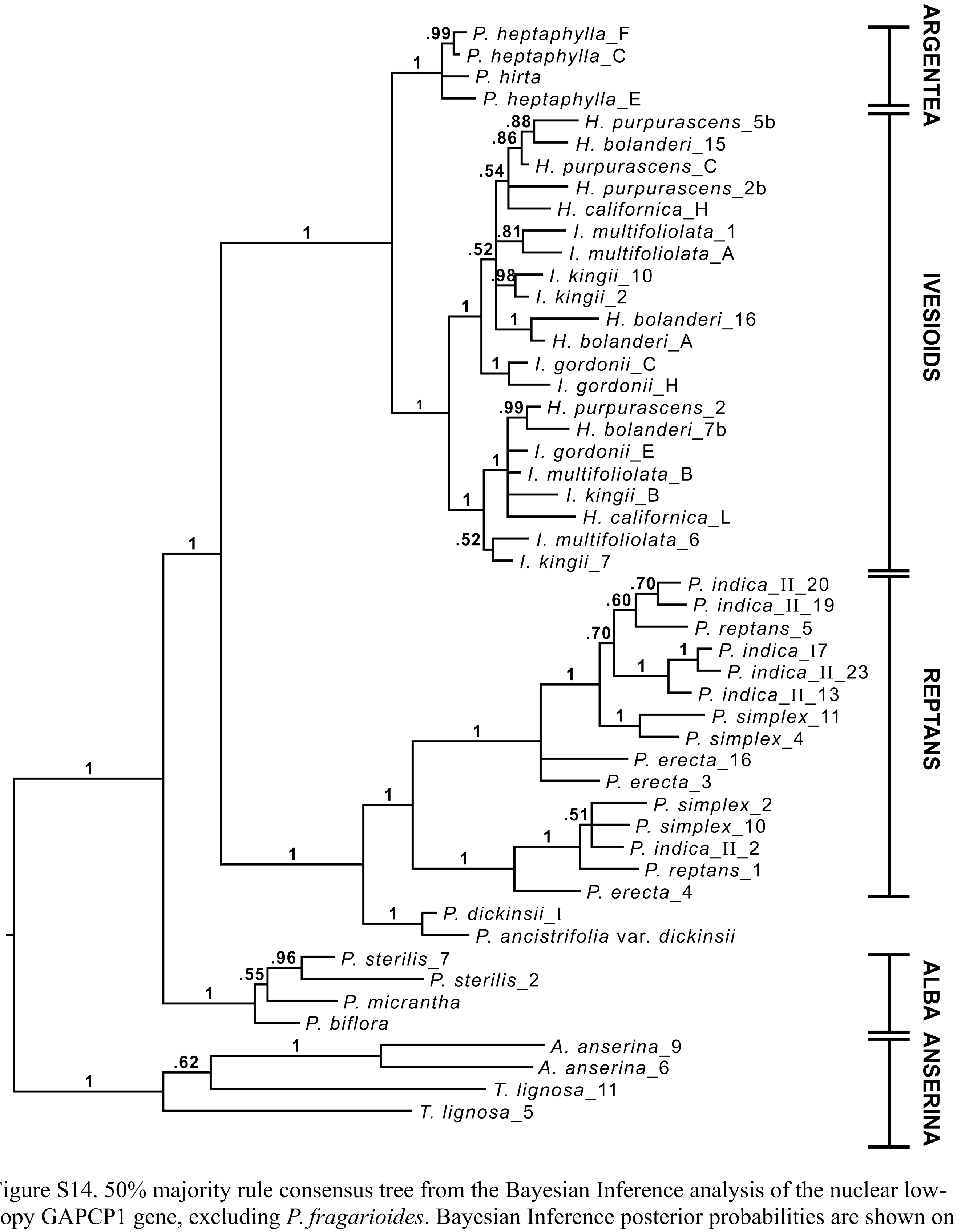

Figure S14. 50% majority rule consensus tree from the Bayesian Inference analysis of the nuclear low-copy GAPCP1 gene, excluding *P. fragarioides*. Bayesian Inference posterior probabilities are shown on the branch above the corresponding nodes. Specific individuals are indicated by Roman numerals and clones are indicated by letters and Arabic numbers. Clade affiliations of species are given to the right, where horizontal lines indicate that the clade is supported (cf. Table 1).

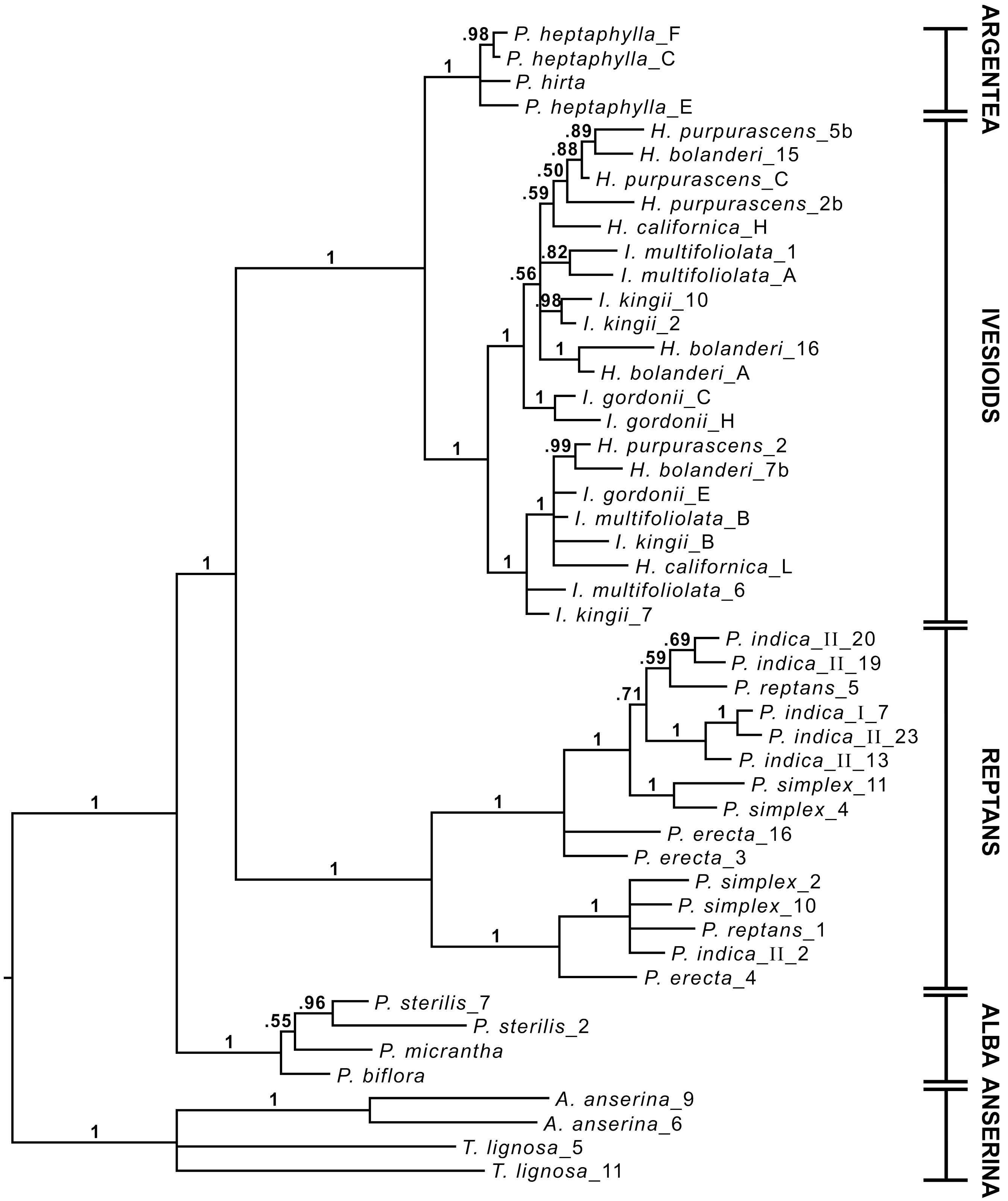

Figure S15. 50% majority rule consensus tree from the Bayesian Inference analysis of the nuclear low-copy GAPCP1 gene, excluding *P. dickinsii* and *P. fragarioides*. Bayesian Inference posterior probabilities are shown on the branch above the corresponding nodes. Specific individuals are indicated by Roman numerals and clones are indicated by letters and Arabic numbers. Clade affiliations of species are given to the right, where horizontal lines indicate that the clade is supported (cf. Table 1).

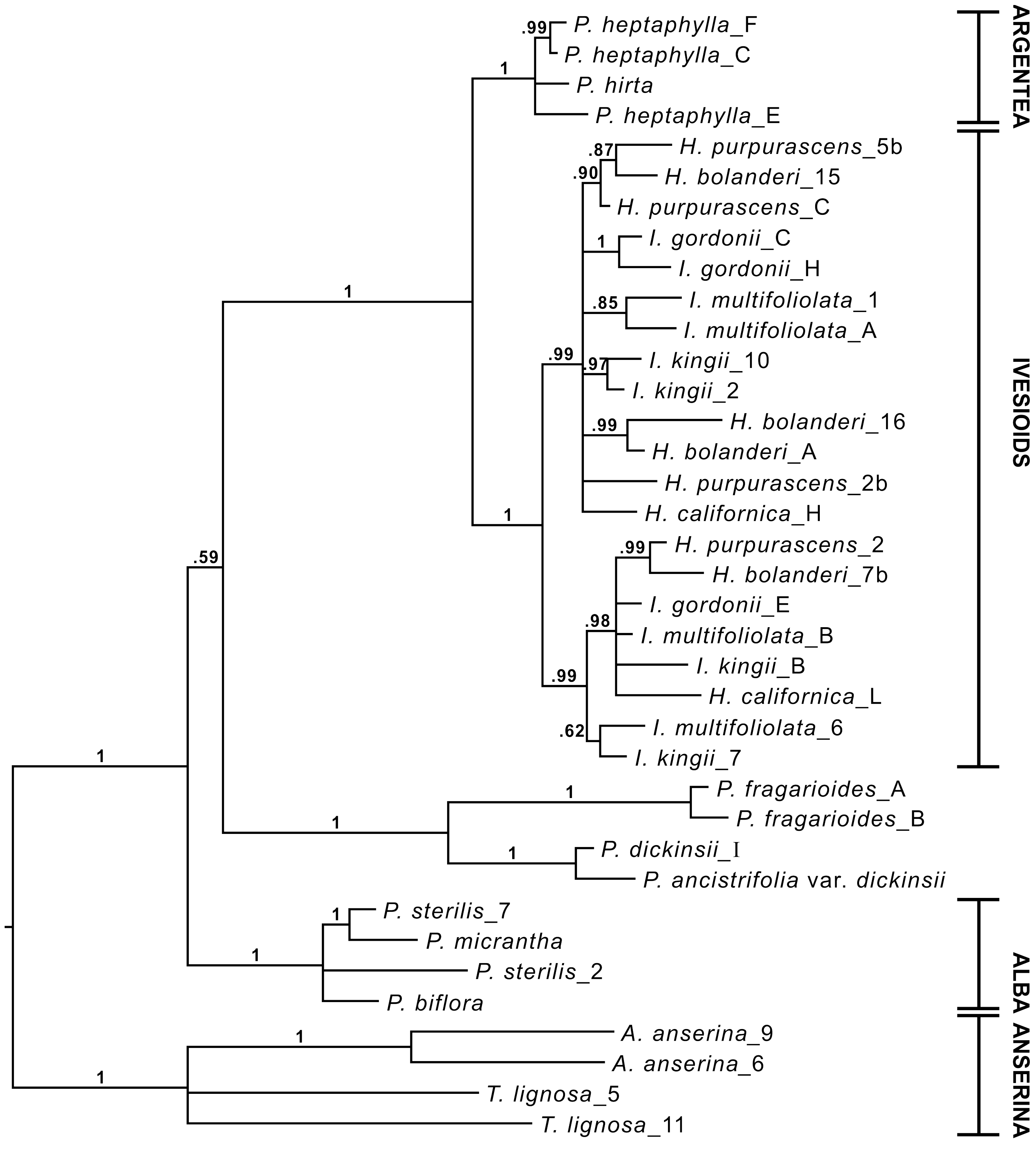

Figure S16. 50% majority rule consensus tree from the Bayesian Inference analysis of the nuclear low-copy GAPCP1 gene, excluding the Reptans clade. Bayesian Inference posterior probabilities are shown on the branch above the corresponding nodes. Specific individuals are indicated by Roman numerals and clones are indicated by letters and Arabic numbers. Clade affiliations of species are given to the right, where horizontal lines indicate that the clade is supported (cf. Table 1).

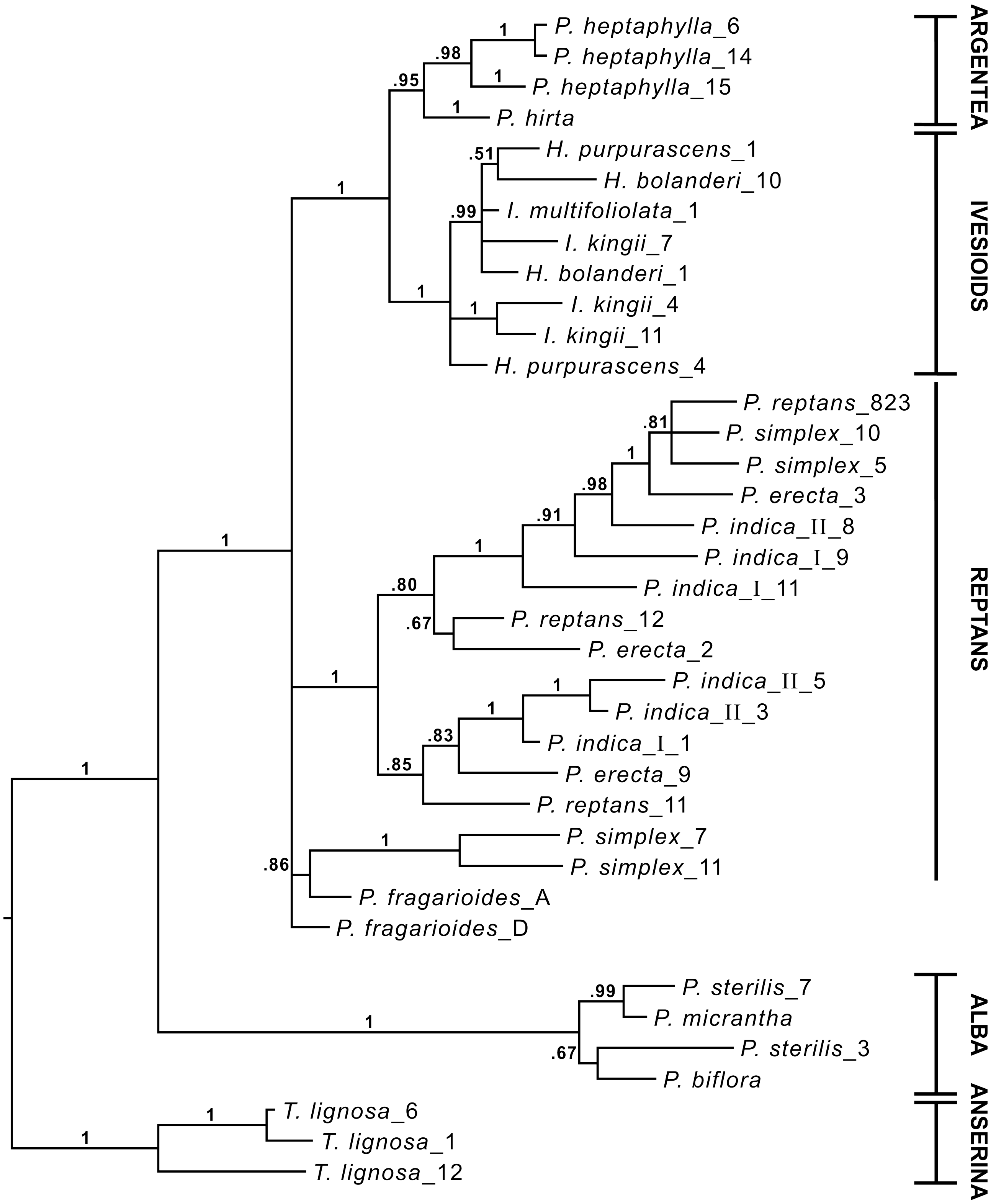

Figure S17. 50% majority rule consensus tree from the Bayesian Inference analysis of the nuclear low-copy GBSSI-2 gene, excluding *P. dickinsii*. Bayesian Inference posterior probabilities are shown on the branch above the corresponding nodes. Specific individuals are indicated by Roman numerals and clones are indicated by letters and Arabic numbers. Clade affiliations of species are given to the right, where horizontal lines indicate that the clade is supported (cf. Table 1).

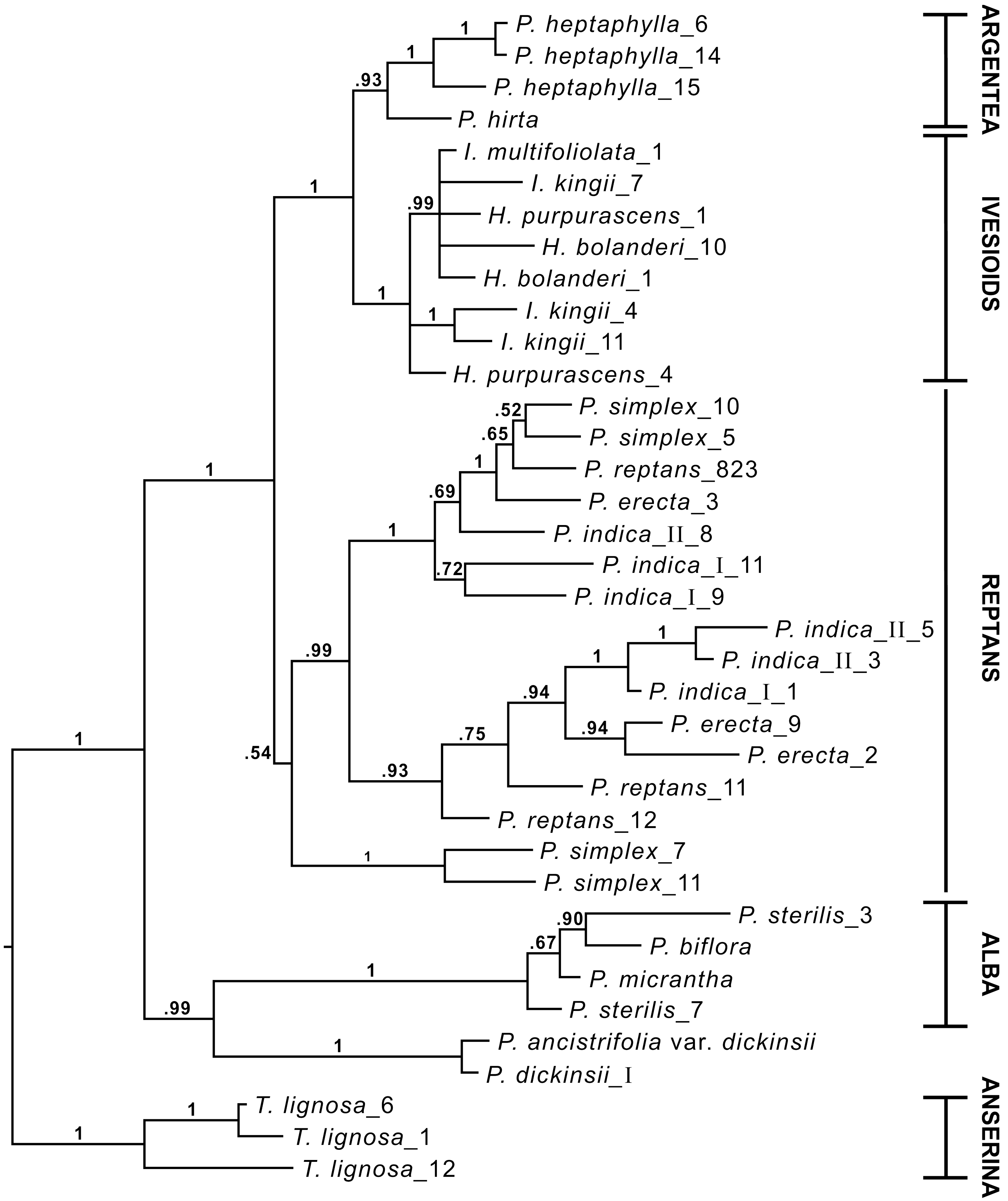

Figure S18. 50% majority rule consensus tree from the Bayesian Inference analysis of the nuclear low-copy GBSSI-2 gene, excluding *P. fragarioides*. Bayesian Inference posterior probabilities are shown on the branch above the corresponding nodes. Specific individuals are indicated by Roman numerals and clones are indicated by letters and Arabic numbers. Clade affiliations of species are given to the right, where horizontal lines indicate that the clade is supported (cf. Table 1).

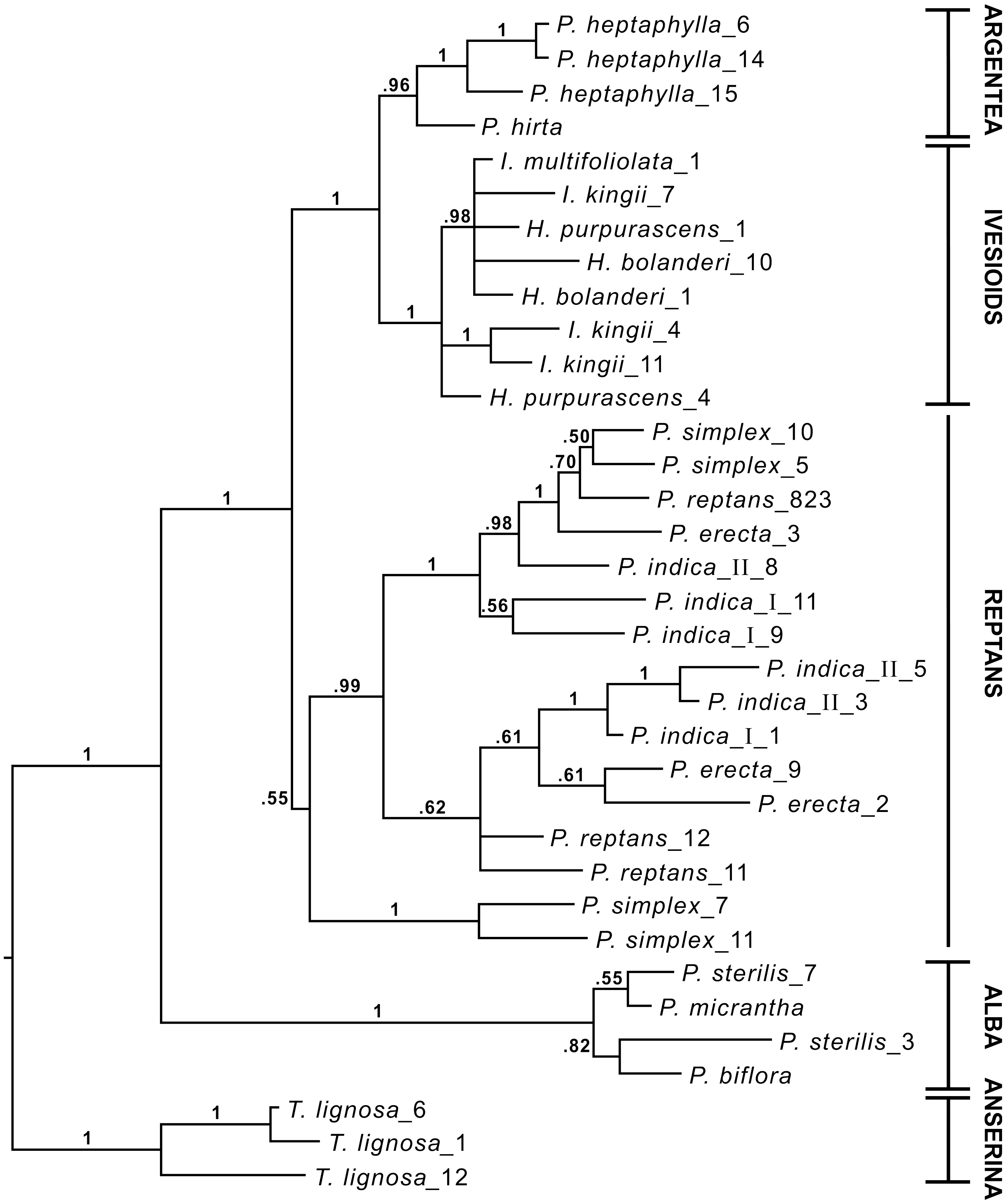

Figure S19. 50% majority rule consensus tree from the Bayesian Inference analysis of the nuclear low-copy GBSSI-2 gene, excluding *P. dickinsii* and *P. fragarioides*. Bayesian Inference posterior probabilities are shown on the branch above the corresponding nodes. Specific individuals are indicated by Roman numerals and clones are indicated by letters and Arabic numbers. Clade affiliations of species are given to the right, where horizontal lines indicate that the clade is supported (cf. Table 1).

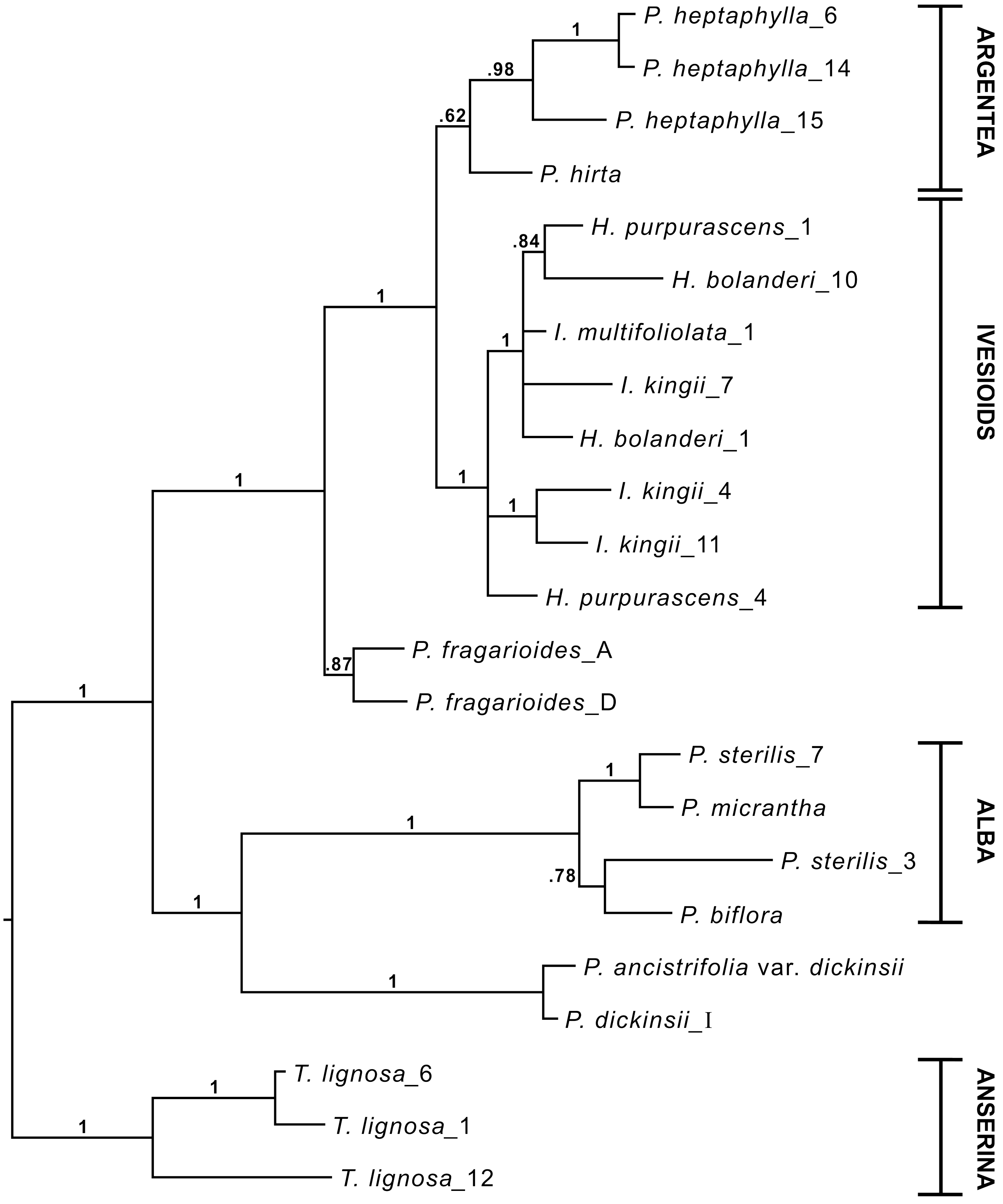

Figure S20. 50% majority rule consensus tree from the Bayesian Inference analysis of the nuclear low-copy GBSSI-2 gene, excluding the Reptans clade. Bayesian Inference posterior probabilities are shown on the branch above the corresponding nodes. Specific individuals are indicated by Roman numerals and clones are indicated by letters and Arabic numbers. Clade affiliations of species are given to the right, where horizontal lines indicate that the clade is supported (cf. Table 1).

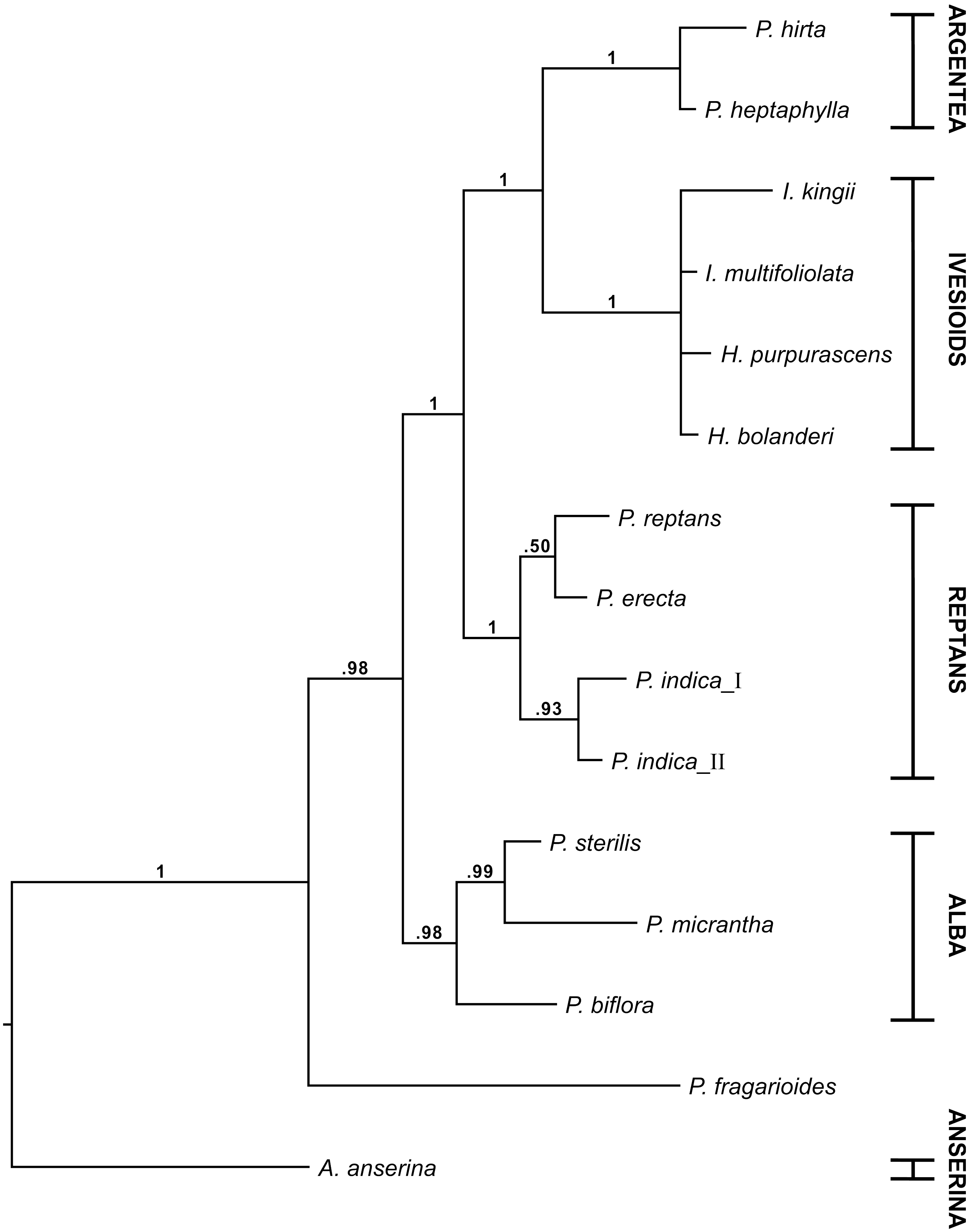

Figure S21. 50% majority rule consensus tree from the Bayesian Inference analysis of the nuclear low-copy SbeI gene, excluding *P. dickinsii*. Bayesian Inference posterior probabilities are shown on the branch above the corresponding nodes. Specific individuals are indicated by Roman numerals and clones are indicated by letters and Arabic numbers. Clade affiliations of species are given to the right, where horizontal lines indicate that the clade is supported (cf. Table 1).

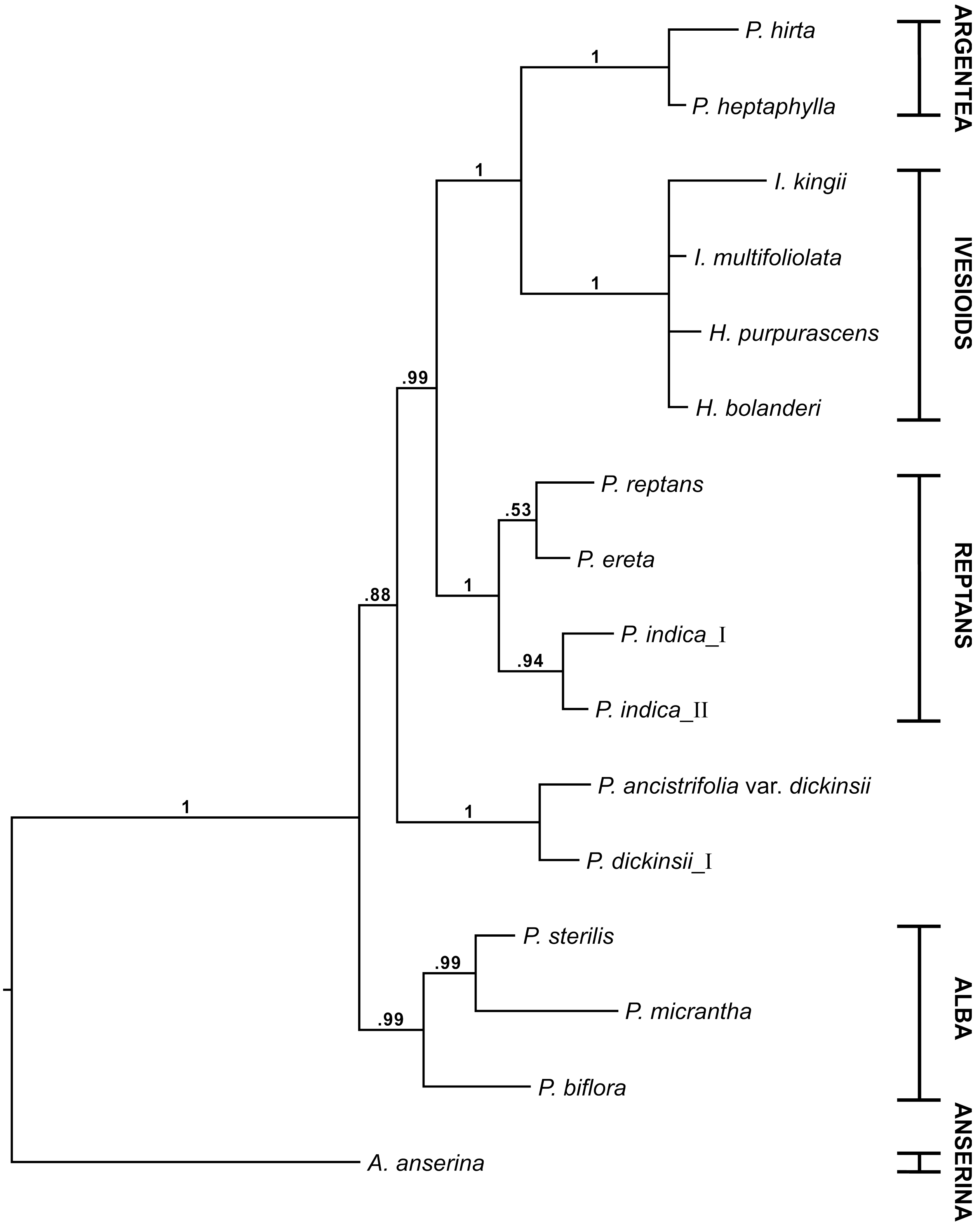

Figure S22. 50% majority rule consensus tree from the Bayesian Inference analysis of the nuclear low-copy SbeI gene, excluding *P. fragarioides*. Bayesian Inference posterior probabilities are shown on the branch above the corresponding nodes. Specific individuals are indicated by Roman numerals and clones are indicated by letters and Arabic numbers. Clade affiliations of species are given to the right, where horizontal lines indicate that the clade is supported (cf. Table 1).

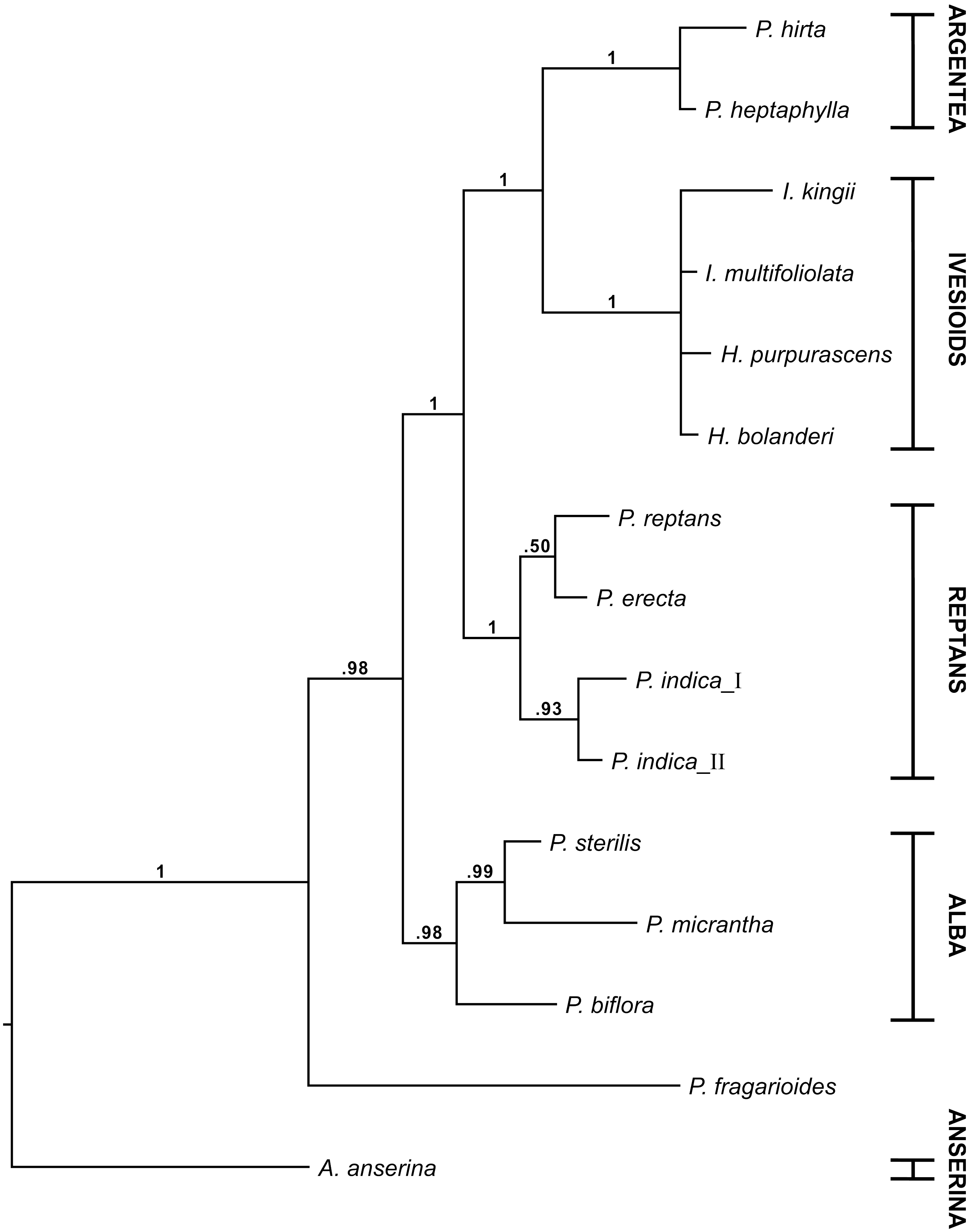

Figure S23. 50% majority rule consensus tree from the Bayesian Inference analysis of the nuclear low-copy SbeI gene, excluding *P. dickinsii* and *P. fragarioides*. Bayesian Inference posterior probabilities are shown on the branch above the corresponding nodes. Specific individuals are indicated by Roman numerals and clones are indicated by letters and Arabic numbers. Clade affiliations of species are given to the right, where horizontal lines indicate that the clade is supported (cf. Table 1).

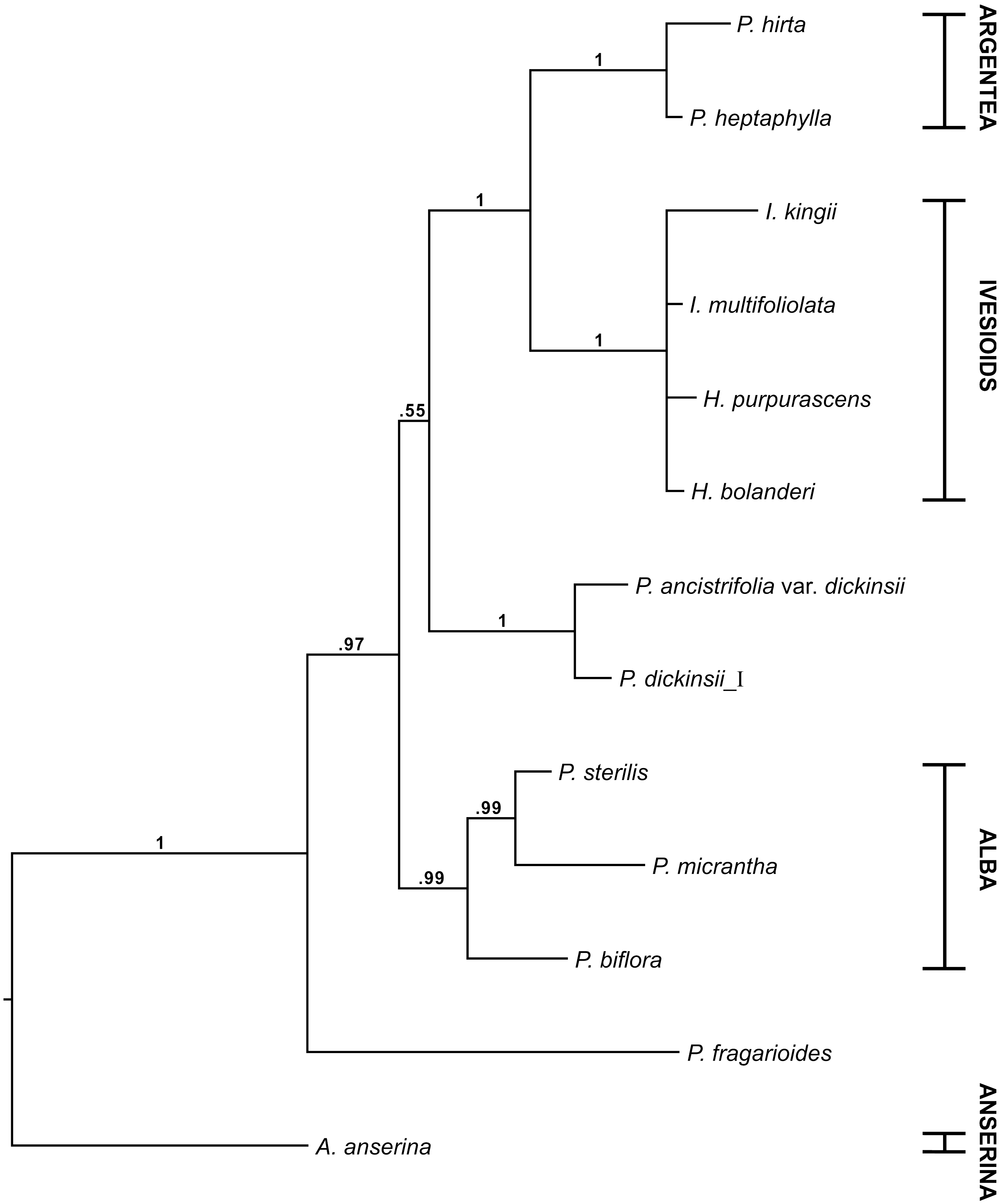

Figure S24. 50% majority rule consensus tree from the Bayesian Inference analysis of the nuclear low-copy SbeI gene, excluding the Reptans clade. Bayesian Inference posterior probabilities are shown on the branch above the corresponding nodes. Specific individuals are indicated by Roman numerals and clones are indicated by letters and Arabic numbers. Clade affiliations of species are given to the right, where horizontal lines indicate that the clade is supported (cf. Table 1).
