# Supplementary material for: Detecting destabilizing species in the phylogenetic backbone of Potentilla (Rosaceae) using low-copy nuclear markers
Source: AoB Plants. 2020 May 9;12(3):plaa017. doi: 10.1093/aobpla/plaa017 (PMC7287270; doi:10.1093/aobpla/plaa017)
Supplement: plaa017_suppl_Supplementary_Table_S2 [file plaa017_suppl_supplementary_table_s2.pdf]

Table S2. Models for the different markers in the Bayesian Inference analyses, as suggested by PartitionFinder2.

**Marker**	**Character set**	**Dataset**				
		all	excl. *P. dickinsii*	excl. *P. fragarioides*	excl. *dick.* and *frag.*	excl. Reptans
matK	1st codon<br>2nd codon<br>3rd codon	GTR	GTR	GTR	GTR	GTR
ITS		GTR+I+G	GTR+G	GTR+G	GTR+I+G	GTR+I+G
DHAR2	1st codon	HKY+G	HKY+G	HKY+G	HKY+I+G	HKY+I+G
	2nd codon	HKY+G	HKY+G	HKY+G	HKY+G	HKY+G
	3rd codon	HKY+G	HKY+G	HKY+G	HKY+G	HKY+G
	introns	GTR+G	GTR+G	GTR+G	GTR+G	GTR+G
GAPCP1	1st codon	HKY+I	HKY+I	HKY+I	HKY	F81
	2nd codon	JC+I	JC+I	JC+I	JC+I	JC
	3rd codon	K80+G	K80+G	K80+G	K80+G	GTR+G
	introns	GTR+G	GTR+G	GTR+G	GTR+G	HKY+G
GBSSI-2	1st codon	F81+G	F81+I	F81+I	F81+I	F81+I
	2nd codon	K80+G	K80+G	K80+G	K80+G	K80
	3rd codon	GTR+I	HKY+G	GTR+I	GTR+I	HKY+I
	introns	GTR+G		GTR+G	GTR+G	
SbeI	1st codon	HKY	HKY	HKY	HKY	HKY
	2nd codon	GTR+I	GTR+I	GTR+I	GTR+I	F81+I
	3rd codon	HKY	HKY	HKY	HKY	HKY
