# Supplementary material for: Detecting destabilizing species in the phylogenetic backbone of Potentilla (Rosaceae) using low-copy nuclear markers
Source: AoB Plants. 2020 May 9;12(3):plaa017. doi: 10.1093/aobpla/plaa017 (PMC7287270; doi:10.1093/aobpla/plaa017)
Supplement: plaa017_suppl_Supplementary_Table_S4 [file plaa017_suppl_supplementary_table_s4.pdf]

Table S4. Log Marginal Likelihood values for analyses in *BEAST. Highest value indicate the prior and clock models best fit to the data.

**Tree prior**	**Clock model**	**Dataset**			
		incl *P. dickinsii* and *P. fragarioides*		excl. *P. dickinsii* and *P. fragarioides*	
		path sampling	steppingstone sampling	path sampling	steppingstone sampling
Birth-Death	Strict	-18129,90	-18132,19	-16906,33	-16907,86
Birth-Death	Relaxed uncorrelated	-18096,33	-18098,53	-16892,76	-16894,17
Yule	Strict	-18139,94	-18141,26	-16931,93	-16933,49
Yule	Relaxed uncorrelated	-18125,79	-18128,13	-16912,12	-16913,45
